# *Shigella* Effector OspB Activates mTORC1 in a Manner That Depends on IQGAP1 and Promotes Cell Proliferation

**DOI:** 10.1371/journal.ppat.1005200

**Published:** 2015-10-16

**Authors:** Richard Lu, Bobby Brooke Herrera, Heather D. Eshleman, Yang Fu, Alexander Bloom, Zhigang Li, David B. Sacks, Marcia B. Goldberg

**Affiliations:** 1 Department of Medicine, Division of Infectious Diseases, Massachusetts General Hospital, Cambridge, Massachusetts, United States of America; 2 Department of Microbiology and Immunobiology, Harvard Medical School, Boston, Massachusetts, United States of America; 3 Department of Laboratory Medicine, National Institutes of Health, Bethesda, Maryland, United States of America; Collège de France, FRANCE

## Abstract

The intracellular bacterial pathogen *Shigella* infects and spreads through the human intestinal epithelium. Effector proteins delivered by *Shigella* into cells promote infection by modulating diverse host functions. We demonstrate that the effector protein OspB interacts directly with the scaffolding protein IQGAP1, and that the absence of either OspB or IQGAP1 during infection leads to larger areas of *S*. *flexneri* spread through cell monolayers. We show that the effect on the area of bacterial spread is due to OspB triggering increased cell proliferation at the periphery of infected foci, thereby replacing some of the cells that die within infected foci and restricting the area of bacterial spread. We demonstrate that OspB enhancement of cell proliferation results from activation of mTORC1, a master regulator of cell growth, and is blocked by the mTORC1-specific inhibitor rapamycin. OspB activation of mTORC1, and its effects on cell proliferation and bacterial spread, depends on IQGAP1. Our results identify OspB as a regulator of mTORC1 and mTORC1-dependent cell proliferation early during *S*. *flexneri* infection and establish a role for IQGAP1 in mTORC1 signaling. They also raise the possibility that IQGAP1 serves as a scaffold for the assembly of an OspB-mTORC1 signaling complex.

## Author Summary

During infection, *Shigella* spp. deliver into the cytoplasm of cells effector proteins that manipulate host cell processes in ways that promote infection and bacterial spread. We have discovered that the *Shigella* effector protein OspB interacts with the cellular scaffolding protein IQGAP1. OspB induces increased cell proliferation by activating mTORC1 kinase, a master regulator of cellular growth, in a manner that depends on IQGAP1. As IQGAP1 has been shown to interact with mTOR and with the mTORC1 activators ERK1/2, we propose that IQGAP1 serves as a scaffold for OspB activation of mTORC1. The presence of OspB and IQGAP1 lead to restricting the area of spread of *S*. *flexneri* in cell monolayers; our data support a model in which the effect of OspB and IQGAP1 on the area of *S*. *flexneri* spread is due to effects on cell proliferation locally within infected foci. As infection of cells and tissue by *Shigella* spp. leads to cell death, increased local cellular proliferation may serve to provide additional protective intracellular niches for the organism within infected tissue.

## Introduction

*Shigella* spp. cause diarrhea and dysentery in humans by invading and spreading through the colonic mucosa. Bacterial invasion of cells, intracellular survival, and aspects of intercellular spread are mediated by bacterial effector proteins delivered into the cell cytoplasm by the *Shigella* type 3 secretion system. Effector proteins interact with host factors to alter cellular processes or cellular signaling cascades in ways that promote infection. *Shigella* infection leads to an acute inflammatory response and abscess formation in the colonic mucosa that is accompanied by death of macrophages, leukocytes, and enterocytes [1–7]. Despite this destruction, bacterial replication within the tissue depends in part on the viability of infected cells. Certain *Shigella* effector proteins promote cell survival. IpgD activates the Akt survival pathway, which delays host cell apoptosis and is associated with an increase in intracellular bacterial replication [8]. OspC3 binds and inhibits caspase-4, which blocks inflammatory cell death [9]. VirA inhibits both necrotic cell death and autophagy [1,10].

The cellular scaffolding protein IQGAP1 participates in the manipulation of the cytoskeleton by *Salmonella* Typhimurium and enteropathogenic *E*. *coli* [11–13]. Here, we demonstrate that IQGAP1 restricts the extent of spread of *S*. *flexneri* in cell monolayers and interacts with the *Shigella* effector protein OspB. OspB has been shown previously to modulate NF-κB activation and phosphorylation of ERK1/2 and activation of cytosolic phospholipase A_2_ and associated IL-8 secretion and transepithelial polymorphonuclear leukocyte migration [14–16]. We show that like IQGAP1, OspB restricts the extent of *S*. *flexneri* spread in cell monolayers. Early during *S*. *flexneri* infection, OspB activates the mechanistic target of rapamycin complex 1 (mTORC1), a central regulator of cell growth and proliferation known to bind IQGAP1 [17,18]. OspB activation of mTORC1 results in increased cell proliferation, dependent on IQGAP1. Increased cell proliferation occurs differentially at infected foci within cell monolayers. These results identify and characterize a targeted mechanism by which *Shigella* manipulates host cell proliferation during infection.

## Results

### The scaffolding protein IQGAP1 limits the area of spread of *S*. *flexneri* in cell monolayers

**Fig 1:**
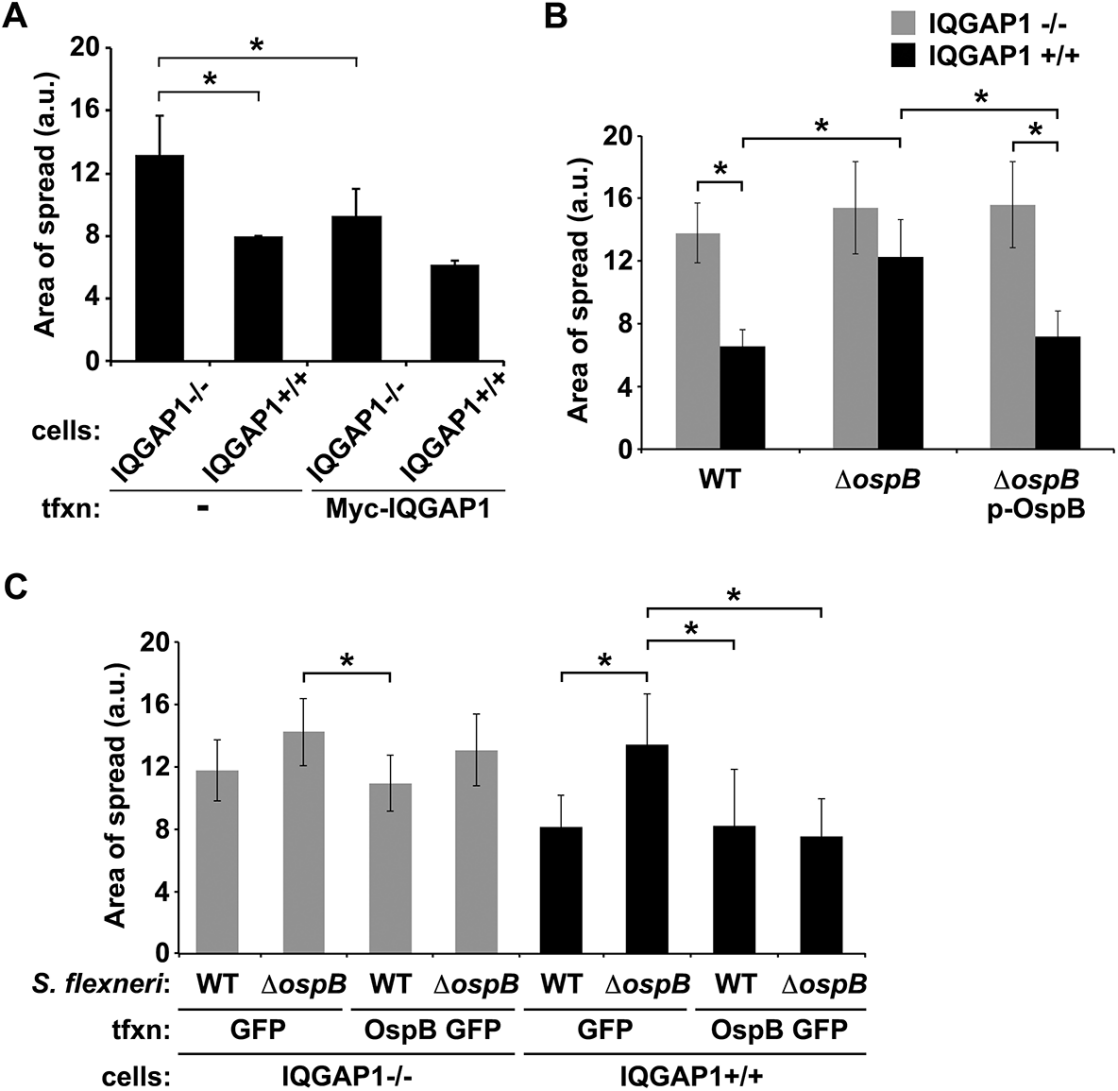
OspB limits *S*. *flexneri* spread dependent on IQGAP1. (A) Area of spread of GFP-producing wild type *S*. *flexneri* in IQGAP1^-/-^ and IQGAP1^+/+^ MEFs, transfected or not with Myc-IQGAP1. a.u., arbitrary units. (B) Area of spread of GFP-producing wild type (WT) or *ospB S*. *flexneri* complemented or not with *ospB* in IQGAP1^-/-^ or IQGAP1^+/+^ cells. (C) Area of spread of GFP-producing WT or *ospB S*. *flexneri* in IQGAP1^-/-^ or IQGAP1^+/+^ cells transfected with a plasmid carrying either *ospB gfp* or *gfp* alone. Mean ± S.D. Data represent three or more independent experiments. *, p < 0.05, Student’s two-tailed t test.

IQGAP1 was selected from a pilot siRNA screen designed to identify human proteins that modulate *Shigella* spread. In this screen, siRNA to IQGAP1 was associated with an increase in the area of wild type *S*. *flexneri* strain 2457T spread through HeLa cell monolayers (IQGAP1 siRNA, 1200 ± 182 a.u. versus control siRNA, 596 ± 42 a.u., p = 0.04, Student’s two-tailed t test), determined by measuring the area of GFP-producing bacteria at individual infectious foci within the monolayer in 384-well format. The impact of IQGAP1 siRNA on area of bacterial spread was validated in 6-well format, where siRNA knock-down of IQGAP1 led to a 1.8-fold increase in area of spread of *S*. *flexneri* (IQGAP1 siRNA, 12 ± 1.4 versus control siRNA, 6 ± 0.4 a.u., p = 0.03, S1 Fig). Upon independently examining the role of IQGAP1 in *S*. *flexneri* spread using monolayers of mouse embryonic fibroblasts (MEFs) that lack or contain IQGAP1, we observed a similar 1.7-fold increase in area of spread in the absence of IQGAP1 (Fig 1A), together suggesting that IQGAP1 might restrict the extent of bacterial spread. Complementation with Myc-IQGAP1 *in trans* significantly reduced the area of spread for IQGAP1^-/-^ MEFs (Fig 1A), indicating that the observed increase in spread in the IQGAP1^-/-^ MEFs was due to the absence of IQGAP1 *per se*.

### The *S*. *flexneri* secreted effector protein OspB binds directly to IQGAP1

**Fig 2:**
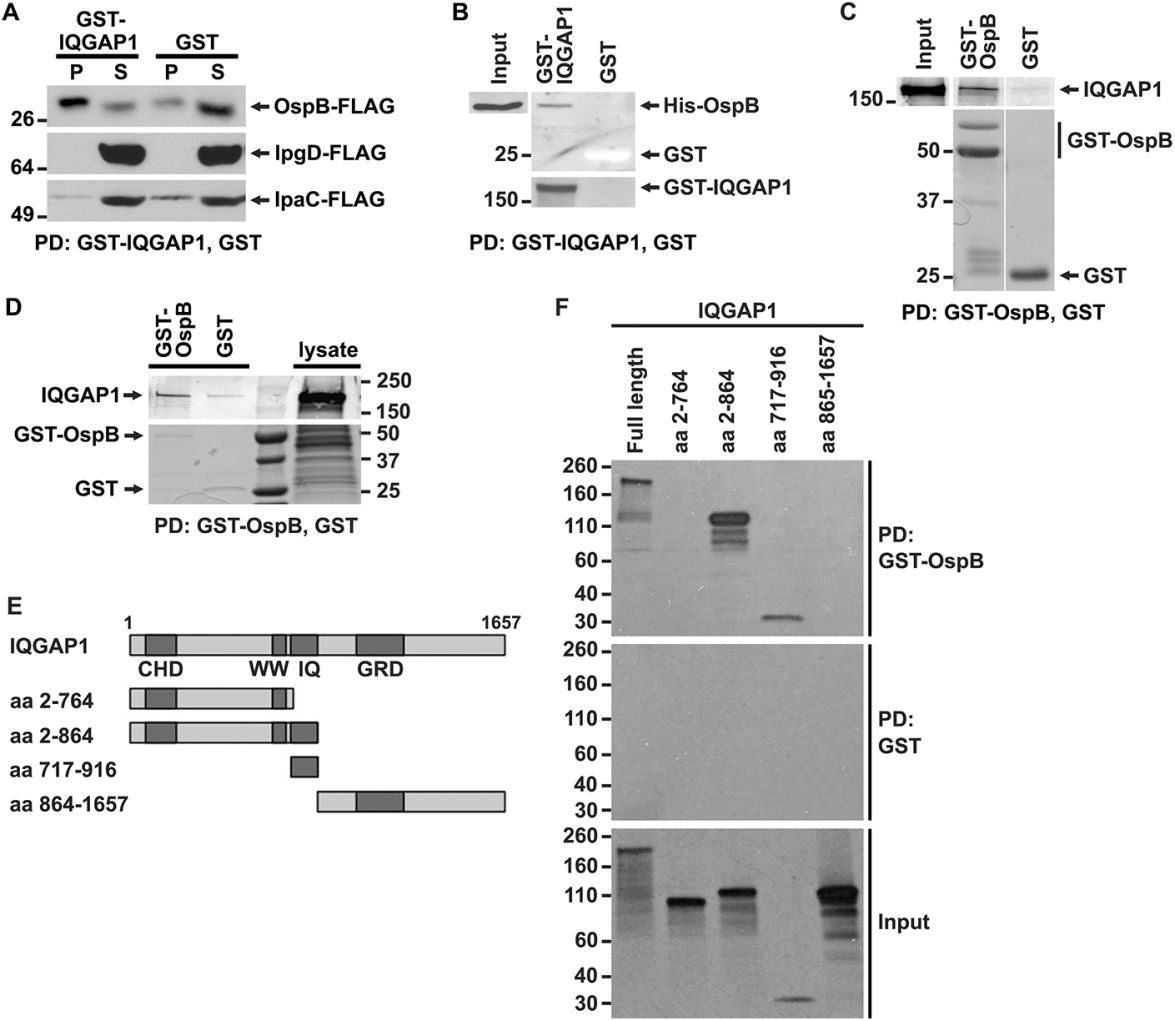
OspB and IQGAP1 reciprocally co-precipitate. (A) GST-IQGAP1 precipitates OspB-FLAG, but not IpgD-FLAG or IpaC-FLAG from *S*. *flexneri* culture supernatants. Western using FLAG antibody. P, precipitated proteins; S, supernatant. (B) GST-IQGAP1, but not GST alone precipitates His-OspB. Each protein was purified from *E*. *coli* (see Materials and Methods). The top two panels are from the same western blot, probed with antibody to His. The bottom panel is a Coomassie stain of the top portion of the same SDS-PAGE gel; GST can be seen as a white band migrating at 25 kD. (C) GST-OspB, but not GST alone precipitates IQGAP1. Each protein was purified from *E*. *coli* (see Materials and Methods). All five panels are from the same gel. After transfer, the top three panels were probed with antibody to IQGAP1, and the bottom two panels were probed with antibody to GST. (D) GST-OspB, but not GST precipitates endogenous IQGAP1 from lysates of MCF-7. GST-OspB and GST were purified from *E*. *coli*. Top and bottom panels are from the same gel. Top panel, western probed with antibody to IQGAP1; bottom panel, Coomassie stained gel. (E) Diagram of IQGAP1 fragments used for mapping site of OspB interaction. CHD, calponin homology domain; WW, polyproline binding region; IQ, IQ region; GRD, Ras GTPase-activating protein-related domain; aa, amino acid. (F) IQGAP1 full length protein and fragments precipitated by GST-OspB, but not by GST alone. GST-OspB and GST were generated in *E*. *coli*, and IQGAP1 constructs were generated as biotinylated proteins by in vitro transcription and translation. Biotinylated proteins were detected with HRP-labeled streptavidin (see Materials and Methods). Data represent three or more independent experiments. Apparent MWs are indicated in Kd.

We interrogated whether IQGAP1 might interact with any of 26 effector proteins translocated by the *Shigella* type 3 secretion system. GST-IQGAP1 pulled down OspB-FLAG from the culture supernatant of *S*. *flexneri* that expressed and secreted it (Fig 2A). Pull down by GST-IQGAP1 was specific, as other secreted proteins, including the effector IpgD and the secreted translocon protein IpaC, were not pulled down (Fig 2A), and was independent of other mammalian proteins, as GST-IQGAP1 used in these experiments had been purified from *E*. *coli*.

To test whether the interaction between OspB and IQGAP1 is dependent on other *S*. *flexneri* proteins, we performed co-precipitation analysis after purifying both proteins from *E*. *coli*. GST-IQGAP1, but not GST alone, pulled down His-tagged OspB (Fig 2B). Moreover, GST-OspB pulled down purified IQGAP1 (Fig 2C), each also purified from *E*. *coli*. OspB also interacts with endogenous IQGAP1 produced in mammalian cells. GST-OspB precipitated IQGAP1 from lysates of MCF-7 human breast epithelial cells, whereas only a faint band was pulled down with GST alone (Fig 2D). Together, these data indicate that OspB binds IQGAP1 specifically and independently of other *S*. *flexneri* or mammalian proteins.

Like many scaffolding proteins, IQGAP1 is a large multidomain protein with multiple binding partners (reviewed in [19]). The N-terminal calponin homology domain (CHD) interacts with F-actin, leading to actin filament bundling and crosslinking in the cell cortex; the polyproline domain (WW) interacts with ERK1, ERK2, and together with upstream sequences, with mTOR; the IQ region interacts with calmodulin, MEK1, MEK2, EGFR, myosin essential light chain, and S100B; the RAS GTPase-activating protein related domain (GRD) interacts with Rac1 and Cdc42; the C-terminal RasGAP domain interacts with βeta-catenin, E-cadherin, CLIP-170, and APC; and the C-terminal Dia1 binding region interacts with Dia1 (reviewed in [19]). To identify the region of IQGAP1 required for interaction with OspB, we generated defined fragments of IQGAP1 in reticulocyte lysates and tested which were pulled down with GST-OspB (Fig 2E and 2F). GST-OspB precipitated the IQ region (residues 717–916) and amino-terminal half (residues 2–864) of IQGAP1, as well as full-length IQGAP1, but not the C-terminal half of the protein (residues 864–1657) or the fragment consisting of residues 2–764, which lacks the IQ region (Fig 2F). These results indicate that the IQ region, which lies between residues 750 and 865, is necessary and sufficient for interactions with OspB.

### OspB contributes to limiting *S*. *flexneri* intercellular spread in a manner that depends on IQGAP1

The observed interaction between IQGAP1 and OspB raised the possibility that OspB might function in IQGAP1 limitation of *S*. *flexneri* spread. In IQGAP1^+/+^ MEFs, an *ospB* mutant displayed significantly increased area of spread compared to wild type *S*. *flexneri*, which was rescued by complementation with *ospB in trans* (Fig 1B, black bars). These differences were absent from IQGAP1^-/-^ MEFs (Fig 1B, gray bars), indicating that OspB function in spread depends on IQGAP1.

These findings were recapitulated when OspB was provided to cells *in trans*. In the presence of IQGAP1, OspB decreased the area of bacterial spread, independently of whether it was delivered by the bacterium or by transient transfection (Fig 1C, black bars), whereas in the absence of IQGAP1, the effect of OspB on spread was muted (Fig 1C, gray bars). We used transient transfection for these experiments because when we attempted to generate cell lines that stably expressed OspB, we found that in the presence of IQGAP1, but not in its absence, introduction of OspB precluded the isolation of stably transfected cells, whereas introduction of vector alone did not. This raised the possibility that the combination of IQGAP1 and OspB perturbed essential cellular pathways. Of note, the efficiency of transient transfection was similar under all conditions. Zurawski et al. [14–16] demonstrated that a *S*. *flexneri ospB* mutant is no difference from WT *S*. *flexneri* in invasion, apoptosis, or the *in vivo* Sereny assay, but is defective in transepithelial polymorphonuclear leukocyte migration. It is notable that these authors did not observe an effect of OspB on plaque size; we postulate that this difference with our results may be because Zurawski et al. performed plaque assays in HeLa cells, in which we also do not observe an OspB-associated phenotype (described below).

As *Shigella* spread through monolayers occurs by actin based motility [20], we compared several parameters relating to this process during infection of IQGAP1^-/-^ versus IQGAP1^+/+^ MEFs, but found only small differences that did not appear to fully explain the observed differences in spread (S1 Table). Given that IQGAP1 plays a role in maintaining the cytoskeleton and thus cell shape and size, it was conceivable that the increase in *S*. *flexneri* spread through cell monolayers observed in the absence of IQGAP1 was simply due to differences in cell size. Alternatively, in the absence of IQGAP1, the dense network of bundled and cross-linked F-actin at the cell cortex might be reduced, which might facilitate *S*. *flexneri* movement into adjacent cells. However, visualization of the actin cytoskeleton and measurements of cell size and cortical actin density showed no difference between IQGAP1^+/+^ and IQGAP1^-/-^ MEFs (S2A Fig and S1 Table).

Intercellular spread of *Shigella* is enhanced by cadherin-cadherin intercellular junctions [21]. Moreover, in polarized Madin-Darby canine kidney II epithelial cells, suppression of IQGAP1 is associated with reduced E-cadherin at intercellular junctions [22]. However, unlike epithelial cells, in MEFs, the classical cadherin that is expressed is N-cadherin [23]. By detecting all cadherins with a pan-cadherin antibody, we found that the distribution and signal intensity of cadherins in the IQGAP1^-/-^ MEFs was indistinguishable from that in IQGAP1^+/+^ MEFs (S2B Fig), suggesting that the observed differences in *S*. *flexneri* area of spread are unlikely to be due to effects of IQGAP1 on cadherin intercellular junctions and that the impact of IQGAP1 on the levels of N-cadherin at intercellular junctions in MEFs is distinct from its impact on levels of E-cadherin at intercellular junctions in epithelial cells. It remains possible that a part of the IQGAP1 phenotype during *S*. *flexneri* infection is due to functions of IQGAP1 that are unrelated to OspB.

### OspB is associated with increased density of cells in the vicinity of plaques

**Fig 3:**
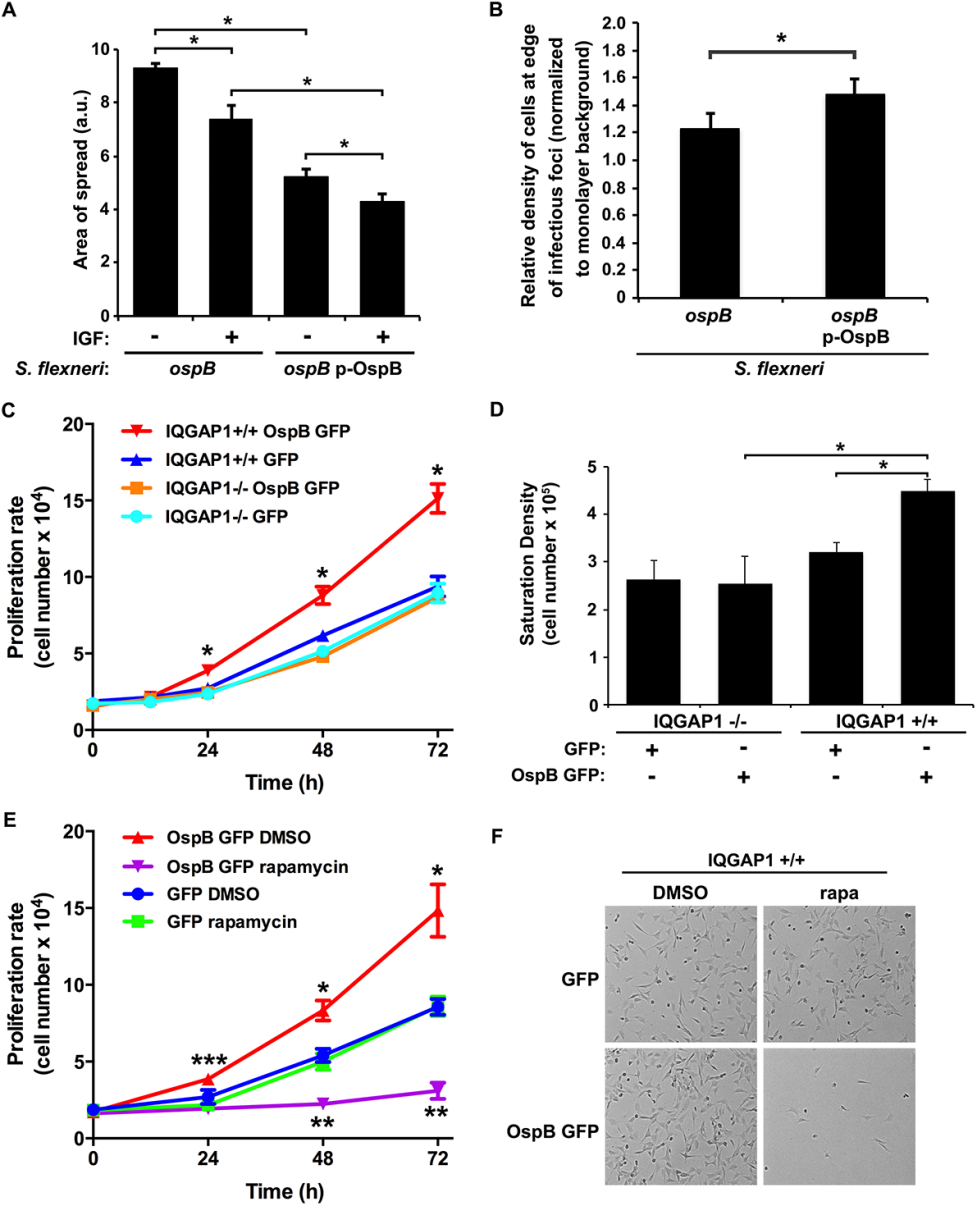
OspB enhances cell proliferation dependent on IQGAP1 and inhibited by rapamycin. (A) Impact of insulin-like growth factor on area of spread of *S*. *flexneri* strains producing or not producing OspB. IGF, insulin-like growth factor. *, p < 0.05, Student’s two-tailed t test. (B) Relative cell density at the edge of infectious foci (normalized to monolayer background) for IQGAP1+/+ cells infected with the *ospB* mutant complemented or not with a plasmid expressing OspB. *, p < 0.05, Student’s one-tailed t test. (C) Proliferation rate of IQGAP1^+/+^ and IQGAP1^-/-^ MEFs transiently transfected with OspB GFP or GFP alone. *, p < 0.05 compared with all other conditions at the same time point, 2-way ANOVA. (D) Saturation density of IQGAP1^+/+^ and IQGAP1^-/-^ MEFs transiently transfected with OspB GFP or GFP alone. *, p < 0.05, Student’s two-tailed t test. (E) Proliferation rate of IQGAP1^+/+^ MEFs transiently transfected with OspB GFP or GFP alone and treated with 10 nM rapamycin or DMSO carrier alone. *, **, p < 0.05 compared with cells transfected with GFP alone at the same time point; ***, p < 0.05 compared with cells treated with rapamycin at the same time point; 2-way ANOVA. (F) Representative images of cells on day 3 of experiment shown in panel E. Data represent mean ± S.D. of three or more independent experiments.

We considered whether the effect of OspB on the area of *S*. *flexneri* spread could be due to effects on cell proliferation. Infection of cells by *Shigella* species results in cell death, which is seen as cellular debris at the center of infectious foci. We reasoned that if cells containing IQGAP1 and infected with *S*. *flexneri* expressing OspB proliferated at an increased rate, then these cells might proliferate sufficiently quickly to replace some of the dying cells within foci of infection, enabling bacteria to spread into these new cells and causing the net area of bacterial spread to be smaller. To assess whether increased cell proliferation *per se* would lead to smaller net area of bacterial spread, we tested the effect of insulin-like growth factor on the area of bacterial spread. The addition of insulin-like growth factor to IQGAP1^+/+^ MEFs led to a 1.3 ± 0.1-fold increase (p = 0.03) in the number of cells over 24 hr. Insulin-like growth factor-induced increase in cell proliferation was associated with a 21% decrease in spread of the *ospB* mutant and an 18% decrease in spread of the *ospB* mutant complemented with OspB (Fig 3A), indicating that increased cell proliferation in the monolayer is associated with decreased area of *S*. *flexneri* spread.

To test whether the presence of OspB was associated with increases in cell proliferation in the vicinity of plaques, we imaged random plaques and random areas away from plaques formed by *S*. *flexneri* producing or not producing OspB at 24 hrs of infection, then blindly comparing the number of nuclei of intact cells within pre-defined grids placed randomly at the edges of infected foci to those within pre-defined grids placed randomly on uninfected areas of the monolayers (see Methods and S3 Fig). The density of cells at the edges of infected foci (normalized to the density of cells in uninfected areas) was significantly more for the OspB-producing strain than for the *ospB* mutant strain (p = 0.03, Fig 3B). Based on these results, we postulate that the observed increase in cell density is sufficient to explain the observed decrease in area of spread.

### OspB enhances cell proliferation in a manner that is enhanced by IQGAP1

To more directly measure the effect of OspB on cell proliferation, the rate of proliferation of non-confluent MEFs transiently transfected with OspB was determined. OspB was associated with significantly increased cell proliferation over 24–72 hrs following plating of the cells, with a slight trend apparent as early as 12 hrs, and this increase depended on IQGAP1 (Fig 3C). The efficiency of cell adhesion to the substrate was indistinguishable among all conditions, and the amount of cell death was low and similar for all conditions (Fig 3C and 3E, and S4A Fig).

A second measure of cell proliferation, the density at which cells attain growth saturation, was increased 1.8-fold (p<0.05) in MEFs transiently transfected with OspB (Fig 3D). Again, this increase depended on IQGAP1. Together, these results establish that OspB enhances cell proliferation in a manner that depends on IQGAP1.

### Increases in cell proliferation are due to OspB activation of mTORC1, which depends on IQGAP1

A central regulator of cell growth and proliferation is the kinase mechanistic target of rapamycin (mTOR). mTOR is a component of two protein complexes, mTOR complex 1 (mTORC1) and mTOR complex 2 (mTORC2), that coordinate cell growth functions in response to growth factors and nutrient availability. Moreover, mTOR interacts with IQGAP1 [17]. Rapamycin, a specific inhibitor of mTOR, completely abolished the increase in proliferation observed in OspB-transfected IQGAP1^+/+^ cells, whereas it had no significant effect on proliferation of cells lacking either OspB or IQGAP1 (Fig 3E and 3F and S4B and S4C Fig). This effect of rapamycin strongly suggested that OspB enhancement of cell proliferation depends on mTOR.

**Fig 4:**
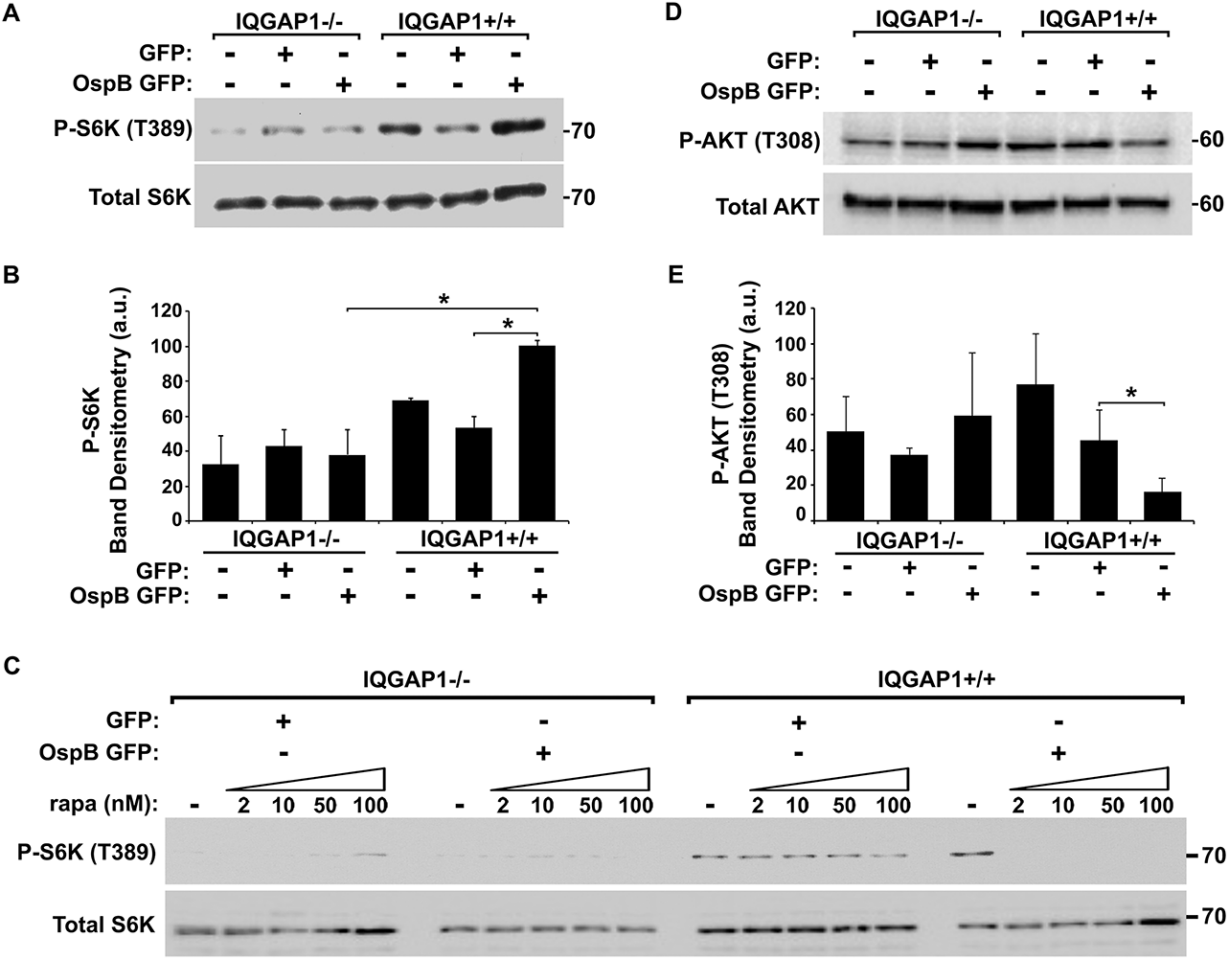
OspB activation of mTORC1. (A-B) Phosphorylation of mTOR substrate S6K in the presence of OspB and dependent on IQGAP1. Representative western blot (A) and band densitometry of phospho-S6K signal normalized to total S6K (B). (C) Inhibition by rapamycin of OspB-induced and IQGAP1-dependent phosphorylation of S6K. (D-E) Reduced phosphorylation of Akt on Thr-308 in the presence of OspB and dependent on IQGAP1. Representative western blot (D), and band densitometry of phospho-Akt Thr-308 signal normalized to total Akt (E). Apparent MWs are indicated in Kd. Densitometry is mean ± S.D. Data represent three or more independent experiments. *, p < 0.05, Student’s two-tailed t test.

We tested whether OspB activated mTOR by examining the phosphorylation state of S6 kinase 1 (S6K), a substrate of mTOR kinase activity that controls cap-dependent translation elongation. Phosphorylation of S6K on Thr-389 was increased 2-fold (p<0.05) in the presence of OspB and in a manner that depended on IQGAP1 (Fig 4A and 4B). Phosphorylation of S6K by OspB was inhibited by rapamycin (Fig 4C). Together, these data establish that OspB activates mTOR.

In IQGAP1^+/+^ MEFs, OspB appeared to sensitize cells to rapamycin. In the presence of OspB, rapamycin dramatically inhibited proliferation of cells, whereas in the absence of OspB, it had no effect (Fig 3E). Moreover, suppression of phosphorylation of the mTOR substrate S6K by rapamycin treatment was enhanced by the presence of OspB (Fig 4C). Detailed analysis of this observation will be explored in future experiments.

Since rapamycin binds and inhibits mTORC1, but not mTORC2 [24,25], our findings suggested that OspB activates mTORC1 and not mTORC2. To further explore this possibility, we examined phosphorylation of Akt at Thr-308, which is inhibited by mTORC1, but not by mTORC2. In the presence of IQGAP1, OspB was associated with a 3-fold decrease in phosphorylation at Akt Thr-308 (Fig 4D and 4E, p<0.05). Activation of mTORC2 increases phosphorylation of Akt at Ser-473 [26], which was unaltered by OspB (S5A Fig). Together, these results are consistent with OspB activating mTORC1 and not mTORC2. Under the conditions used, GST-OspB did not precipitate mTOR from MCF-7 cell lysates.

Akt is itself an upstream regulator of mTORC1 activity and a binding partner of IQGAP1 [27]. This raised the possibility that, even though Akt Thr-308 phosphorylation was decreased by OspB (Fig 4D), OspB might activate mTORC1 via Akt. To directly test this, we examined activation of mTORC1 in the presence of the phosphatidylinositol 3-kinase inhibitor LY294002, which inhibits Akt activation. In the presence of LY, infection with wild-type *S*. *flexneri* induced phosphorylation of S6K, yet phosphorylation of Akt was inhibited (S5B Fig), indicating that mTORC1 is activated independently of Akt.

### OspB activates mTOR during *S*. *flexneri* infection

**Fig 5:**
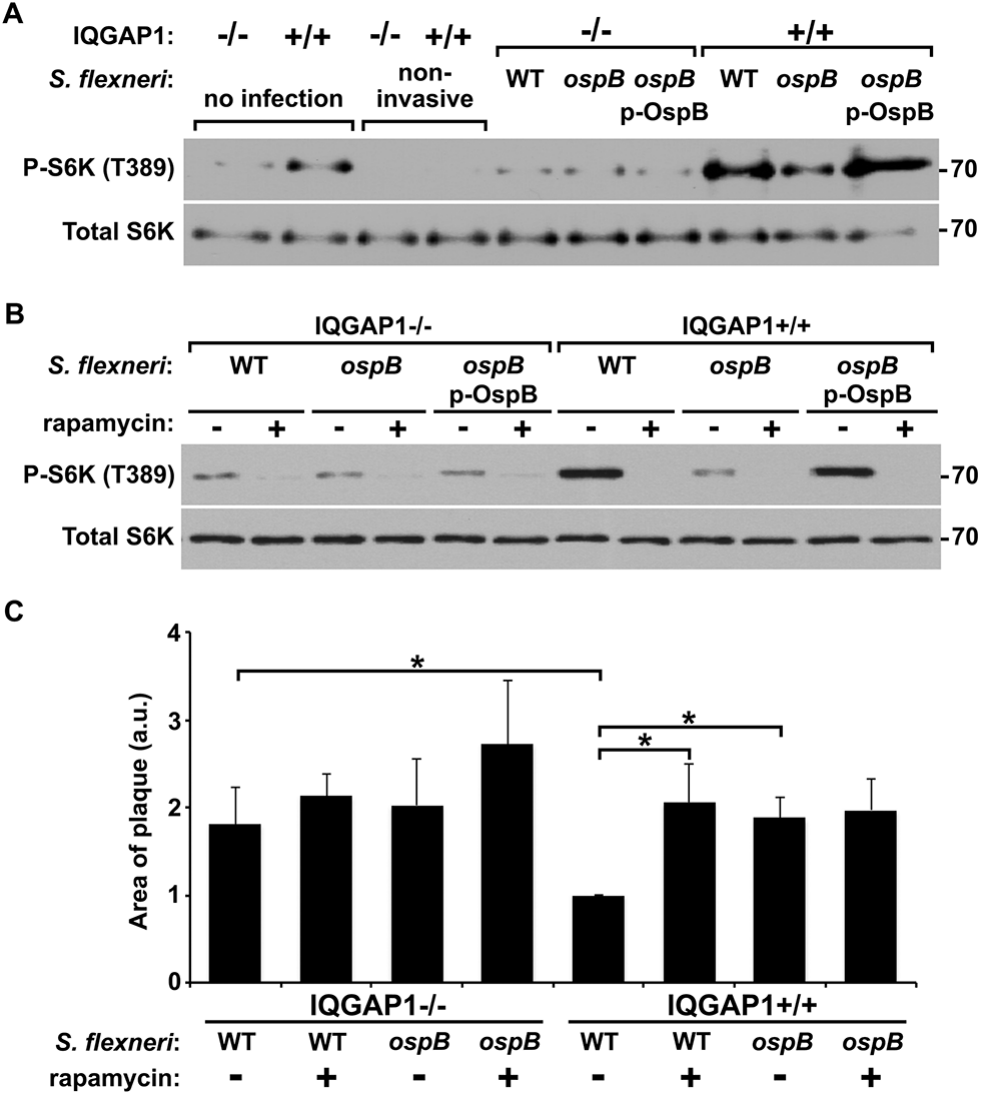
OspB activates mTOR during *S*. *flexneri* infection. (A-B) Phosphorylation of S6K during infection of IQGAP1^-/-^ and IQGAP1^+/+^ MEFs with non-invasive, wild type (WT), Δ*ospB* (*ospB*), or Δ*ospB* complemented with p-*ospB* (*ospB* p-OspB) *S*. *flexneri* (A), and in the presence or absence of 10 nM rapamycin (B). Apparent MW are in Kd. (C) Area of plaques formed in monolayers by wild type or Δ*ospB S*. *flexneri* in the presence or absence of 10 nM rapamycin. Data represent mean ± S.D. of three or more independent experiments. *, p < 0.05, Student’s two-tailed t test.

To test the relevance of OspB activation of mTOR during *S*. *flexneri* infection, we compared S6K phosphorylation in cells infected with an *ospB* mutant to that in cells infected with WT *S*. *flexneri* or the complemented *ospB* mutant. At 1 hr of infection of IQGAP1^+/+^ MEFs, WT or *ospB* complemented *S*. *flexneri* induced more phosphorylation of S6K than the *ospB* mutant (Fig 5A), indicating that OspB delivered by *S*. *flexneri* activates mTOR early during infection. Activation of mTOR during infection depended on IQGAP1, since S6K phosphorylation remained at baseline in IQGAP1^-/-^ MEFs. For cells that were exposed to the non-invasive *S*. *flexneri* mutant BS103, S6K phosphorylation was lower than baseline, suggesting that the presence of extracellular bacteria might suppress mTOR activation, perhaps by reducing the concentration of amino acids in the extracellular media [28]. Treatment of cells with rapamycin inhibited OspB+ *S*. *flexneri*-induced phosphorylation of S6K (Fig 5B), indicating that S6K phosphorylation was mediated by mTOR kinase. Rapamycin had no effect on bacterial growth *in vitro* (S6A Fig).

Tattoli *et al*. described that *S*. *flexneri* inhibits mTOR at 4 hrs of infection via amino acid starvation [29]; these authors did not observe the initial activation of mTOR at 1 hr of infection that we describe here. To explore these differences, we compared mTOR activation at 1 and 4 hrs. of infection of HeLa cells, which were used by Tattoli *et al*., as well as of the intestinal epithelial cell line Caco2 and of MEFs (S6B Fig). Consistent with the findings of Tattoli *et al*., at 4 hrs, mTOR signaling in each of the three cell lines was inhibited by *S*. *flexneri* (S6A Fig). At 1 hr, mTOR activation was observed in Caco2 cells and MEFs, but was not apparent in HeLa cells. The absence of initial activation in HeLa cells appeared to be due to a high level of mTOR activation at baseline (i.e., in uninfected cells) that was not present in the other cell lines. This baseline level of mTOR activation might result from the genetic background of this cancer cell line or have been acquired subsequent to its initial isolation. Taken together, these findings suggest that in cells that display at baseline a low level of mTOR activity, *S*. *flexneri* infection induces dynamic changes of mTOR activation.

### Inhibition of mTOR is associated with increased area of spread, dependent on IQGAP1

To test whether the increase in *S*. *flexneri* area of spread that we observed in cells lacking IQGAP1 or with strains lacking *ospB* (Fig 1) might be due to decreased mTOR activation, we measured spread in the presence or absence of rapamycin. The presence of rapamycin was associated with an increase in the area of spread of wild-type bacteria to that of the *ospB* mutant (Fig 5C). Rapamycin had no effect on the spread of the *ospB* mutant, indicating that a rapamycin sensitive pathway is responsible for the effect of *ospB* on spread. Moreover, the effect of rapamycin on the spreading ability of the wild type strain was dependent on IQGAP1. These results are consistent with IQGAP1-dependent activation of mTOR by OspB causing changes in cell proliferation and growth that result in a decrease in area of spread of *S*. *flexneri* through cell monolayers.

## Discussion

The mTOR complexes are protein kinases that serve as master regulators and coordinators of cellular growth and metabolism in response to extracellular and intracellular growth factors and nutrient states. Deregulation of the mTOR signaling pathway plays a central role in the transformation and the uncontrolled growth of numerous cancers, as well as roles in diabetes, obesity, neurodegeneration, and aging [30]. In this study, we demonstrate that the *Shigella* type 3 secreted effector protein OspB activates mTOR activity during cellular infection. Bacterial type 3 secreted effectors alter diverse cellular pathways; to our knowledge, this is the first description of a type 3 effector protein that activates mTOR and thereby promotes host cell survival.

mTOR exists in two physically distinct multi-protein signaling complexes, mTORC1 and mTORC2 [30,31]. The two complexes share the core kinase mTOR, as well as DEPTOR, mLST8, TTI1, and TEL2. mTORC1 is defined by also containing regulatory associated protein of mTOR (RAPTOR), whereas mTORC2 instead contains rapamycin-insensitive companion of mTOR (RICTOR). The two complexes are generally activated by different sets of signals, and their activation leads to distinct, yet overlapping cellular responses. OspB activation of mTOR appears to be specific for the mTORC1 complex, as OspB-mediated activation is completely inhibited by short-term treatment with the specific mTORC1 inhibitor rapamycin and is associated with decreased phosphorylation of Akt on Thr-308 (Fig 4). The kinase activity of activated mTORC1 leads to increases in cellular protein synthesis, metabolism, and growth, and to inhibition of autophagy. Indeed, activation of the Akt-mTOR pathway by *Salmonella typhimurium* in macrophages attenuates the autophagic response [32].

Our data support a model in which the effect of IQGAP1 and OspB on the area of *S*. *flexneri* spread through cell monolayers is due to effects on cell proliferation (S7 Fig). Infection of cells by *Shigella* species results in cell death, which is seen as cellular debris at the center of infectious foci. We propose that soon after initial entry into a cell (either from the extracellular milieu or via bacterial spread from an infected adjacent cell), OspB activates mTORC1, thereby triggering a cellular pathway that leads to increased cell proliferation in the infected cell and possibly in adjacent cells. Cellular proliferation is sufficient to replace some of the dying cells within foci of infection and results in increased numbers of viable cells within the area of infection and increased density of cells within the focus of infection. Since the length of time the bacterium spends spreading from one cell into the adjacent cell (the time within the protrusion and the new vacuole) is substantial—similar to the length of time the bacterium spends in the cell cytosol prior to protrusion formation [33]—the presence of increased numbers of cells within the immediate area of infection slows the net lateral movement of the bacteria through the monolayer. The net effect is that the area of bacterial spread within the monolayer is reduced compared with conditions that lack OspB or IQGAP1. Multiple findings presented here support this model: (1) OspB activation of mTORC1 is associated with increased cell proliferation (Fig 3). (2) Growth factor-induced increases in cell proliferation are associated with decreased area of *S*. *flexneri* spread (Fig 3). (3) The area of *S*. *flexneri* spread is decreased by the presence of OspB and IQGAP1 (Fig 1) and is increased by the addition of rapamycin in a manner that depends on OspB and IQGAP1 (Fig 5C), indicating that the observed decreases in area of spread are due to IQGAP1-dependent OspB activation of mTORC1. (4) OspB induces an increase in cell density at the edges of infected foci (Fig 3B), indicating that the effect of mTORC1 activation on cell proliferation in infected monolayers is observed locally within infected foci. Increased local cellular proliferation may serve to provide additional protective intracellular niches for the organism in areas of cell death within infected tissue.

These findings do not exclude the possibility that another effect of mTORC1 activation on cellular function contributes to the effect of OspB and IQGAP1 on area of spread or that mTORC1 activation has other beneficial effects on *Shigella* infection. A related consideration is that OspB activates NF-κB, a critical cell survival signal [16]. Since the effect of OspB on *S*. *flexneri* spread was completely blocked by rapamycin (Fig 5C), the spread phenotype appears to be primarily or exclusively due to mTORC1 activation, rather than NF-κB induced cell survival. Also consistent with this is our observation that OspB had no effect on cell survival *per se* (S4A Fig).

Activation of mTORC1 is observed at 1 hr of infection, with a subsequent decrease in SK6 phosphorylation by 4 hrs of infection (S5 Fig and [29]). Despite the transient nature of S6K phosphorylation, increases in cell proliferation and cell density are observed as early as 24 hrs (Fig 3C). A possible explanation for these findings is that transient activation of mTORC1 is sufficient to trigger a cell proliferation pathway that is sufficiently active to significantly impact the number of cells. Alternatively, mTORC1 activation may be bimodal within the infected cells in the monolayer, dependent on the length of time individual cells have been infected, i.e., in cells that were infected recently, due either to bacteria entering from the extracellular milieu or to bacterial spread from infected adjacent cells, mTORC1 would be activated, and in cells that have been infected for 4 hrs or longer, mTOR would be inhibited. At any given time, the level of mTORC1 activation in the monolayer as a whole would reflect the mix of all cells, yet proliferation would be activated in all recently infected cells. Consistent with this possibility is our observation that OspB induces increased cell density locally within infected monolayers (Fig 3A and 3B).

OspB activation of mTORC1 is enhanced by the scaffolding protein IQGAP1 (Fig 4), and OspB interacts directly with IQGAP1 independent of other *S*. *flexneri* or mammalian proteins (Fig 2). IQGAP1 links components of multiple cellular signaling pathways [34]. Our findings raise the possibility that, in addition to its previously described roles, IQGAP1 serves as a scaffold for OspB activation of the mTORC1 signaling complex. Our data suggest that the IQ motif of IQGAP1 is required and sufficient for its interaction with OspB (Fig 2E and 2F); the IQ motif is immediately C-terminal to the WW region of IQGAP1 implicated in interactions with mTOR [17], raising the possibility that OspB and mTOR are in close proximity when bound to IQGAP1 (S7 Fig). Among known activators of mTORC1 is the MEK/ERK MAP kinase signaling pathway, as ERK inactivates the TSC complex, which inhibits mTORC1 [35]. IQGAP1 is known to bind directly to MEK1/2 and ERK1/2, modulating their activation [36–38], and to interact with mTOR [17,18]. Of note, the *Shigella* type 3 secreted effector OspF irreversibly inactivates MAP kinases, including ERK1/2 [39]. However, other data suggest that during infection, OspB is required for maximal phosphorylation of ERK1/2 [14,15], raising the possibility that OspB activation of mTORC1 might occur via ERK1/2. How OspB activation of ERK1/2 and subsequent ERK1/2-dependent activation of mTORC1 might be reconciled with OspF inactivation of ERK1/2 is at present unclear.

Whereas OspB restriction of *S*. *flexneri* spread is markedly enhanced by IQGAP1 (Fig 1), in the absence of IQGAP1, when present both *in trans* and delivered by bacterial type 3 secretion, OspB has an effect on bacterial spread (Fig 1C, left). This is mirrored by a slight trend, albeit statistically insignificant, of OspB increasing cell proliferation via mTORC1 in the absence of IQGAP1 (S4B and S4C Fig, OspB GFP plus DMSO versus OspB GFP plus rapamycin). Taken together, these data suggest that when high levels of OspB are present, a small effect on mTORC1 may occur even in the absence of IQGAP1. This is consistent with our model that IQGAP1 is a platform for OspB activation of mTORC1. In this model, when OspB is present in low concentrations, IQGAP1 is critical for promoting OspB activity, perhaps by localizing it optimally and/or bringing it into close proximity to its target; in contrast, when OspB is abundant, it can interact with its target even if not bound to IQGAP1.

Although additional work will be required to determine the mechanism of OspB activation of mTORC1, our data provide additional evidence in support of a scaffolding role for IQGAP1 upstream of mTORC1 signaling. Roles for IQGAP1 as a scaffold in signaling by bacterial pathogens have been proposed previously [11,12,40]. Together with our findings that *Shigella* OspB depends on IQGAP1 for activation of mTOR, we speculate that IQGAP1 has been hijacked as a signaling scaffold by multiple pathogens.

OspB activation of mTORC1 was completely blocked by rapamycin. Rapamycin, in complex with FK506-binding protein of 12 kDa (FKBP12), inhibits mTORC1 by binding to the FKB12-rapamycin-binding (FRB) domain of mTOR [41]. Recent structural studies suggest that this interaction reduces accessibility of substrates to the catalytic cleft of mTOR [42]. Our observation that OspB appears to sensitize cells to the inhibitory effect of rapamycin (Figs 3E and 4C) raises the possibility that OspB promotes the interaction or the specific activity of rapamycin with respect to mTORC1; ongoing studies are investigating these possibilities.

## Methods

### Bacterial strains, plasmids, growth conditions

Wild-type *S*. *flexneri* serotype 2a strain 2457T [43], an isogenic *ospB* deletion mutant strain (gift from C. Lesser), and an isogenic non-invasive strain, BS103, which is cured of the virulence plasmid [44], were grown in tryptic soy broth from individual colonies that were red on agar containing Congo red.

### Generation of IQGAP1 and OspB constructs

IQGAP1 expression plasmids have been described [45]. For bacterial expression, *ospB* was cloned under the control of the native promoter in pACYC184, and for mammalian expression, into pcDNA3 as a transcriptional fusion to *gfp*. Each of 26 *S*. *flexneri* type III secreted effector proteins and the two secreted translocon pore proteins IpaB and IpaC were tagged at the C-terminus with FLAG and expressed under the control of the impaired Tac promoter in pDSW206 [46], as described [47]. The coding sequence for *ospB*::*flag* was amplified by PCR as a BamHI-EcoRI fragment and inserted into pGEX2T or pRSET-A at BamHI and EcoRI sites. The sequences of all plasmids were verified by DNA sequencing.

### Cells

HeLa cells (ATCC), Caco2 (ATCC), and MCF-7 (gift of A. Dutta) cells were maintained in Dulbecco’s modified media (DMEM) supplemented with fetal bovine serum (10% vol/vol). IQGAP1^-/-^ and IQGAP1^+/+^ MEFs had been previously isolated from IQGAP1^-/-^ and littermate control IQGAP1^+/+^ mice as described [38]; all MEFs used for experiments were early passage.

### Transfection and siRNA

Cells, seeded in 6-well plates, were transfected with 1.5–5.0 ug plasmid DNA using Lipofectamine (Invitrogen, Carlsbad, CA), per manufacturer’s directions. Transfected cells were infected 16–72 hr later. Transfection efficiency was determined by quantification of cells expressing GFP in transfections performed in parallel by microscopic and FACS analysis. The transfection efficiency of the OspB GFP plasmid was comparable to that of the GFP plasmid. siRNA targeting IQGAP1 (SMARTpool M-004694-01) was from Dharmacon RNAi Technologies (Thermo Fisher Scientific). Reverse transfection of 8 nM siRNA into HeLa cells was performed with HiPerFECT (Qiagen) according to the manufacturer instructions.

### Analysis of cell morphology

Cell area of IQGAP1^-/-^ and IQGAP1^+/+^ MEFs at 50% and 100% confluency was calculated on images of monolayers that had been fixed and stained with phalloidin using IP Lab software. The density of cortical actin was assessed on fixed and phalloidin-stained monolayers at 30% confluence by calculating the ratio of fluorescence in 4 μm x 4 μm regions of the cell cortex to that of total fluorescense of the cellular region on images captured at 225x magnification.

### Analysis of cell proliferation rate

The rate of cell proliferation was determined by measuring serum-independent growth of transiently transfected cells. Transfection with the OspB GFP or GFP construct was performed in DMEM without fetal bovine serum. The next day, the cells were trypsinized and seeded at 2x10^4^ per well in 12-well plates in DMEM supplemented with 10% fetal bovine serum (vol/vol) for 4 hrs to allow the cells to attach. Thereafter, the medium was replaced with DMEM supplemented with 1% fetal bovine serum (vol/vol). Time zero was defined as 4 hrs after plating; at the indicated times (12 hrs and 1, 2, and 3 days), the cells were washed twice with PBS, trypsinized, and counted using a hemocytometer.

### Analysis of cell saturation density

The saturation density of cells was determined as above, except that after the 4-hr attachment period, the medium was supplemented with 10% fetal bovine serum (vol/vol), and the cells were counted once on day 3.

### Analysis of mTOR activation

Cells were maintained in DMEM supplemented with 10% fetal bovine serum (vol/vol). Twenty-four to 48 hrs after transfection with the OspB GFP or GFP construct, cells were washed once with PBS and harvested for western blot analysis. Where appropriate, rapamycin was added to 10 nM (unless otherwise indicated) for 1 hr before washing and harvesting. Where appropriate, LY294002 (50 μM, Cell Signaling, #9901S) was added immediately prior to addition of bacteria.

### Bacterial cell infection assays

Cells seeded in 6-well plates were routinely infected at a multiplicity of infection (MOI, bacteria:cell) of 50–150 with exponential phase *S*. *flexneri*. The infected plates were centrifuged at 830 *g* for 10 min and were returned to 37°C for 1 hr. Where appropriate, rapamycin (to 10 nM) or DMSO carrier alone was added to the monolayers at the same time as the bacteria were added.

For the analysis of actin tail formation and the formation of cell surface protrusions, after 1 hr of infection, gentamicin (25 μg/ml), which kills extracellular but not intracellular *Shigella*, was added to the media. The infection was allowed to proceed for an additional 1 hr, at which time, the cells were fixed in 3.7% paraformaldehyde in F buffer (5 mM PIPES pH 7.2, 5 mM KCl, 137 mM NaCl, 4 mM NaHCO_3_, 0.4 mM KH_2_PO_4_, 1.1 mM Na_2_HPO_4_, 2 mM MgCl_2_, 2 mM EGTA, 5.5 mM glucose), permeabilized in 0.5% Triton X-100 in F buffer (20 min, room temperature), and blocked in 0.1 M glycine in F buffer (10 min, room temperature). Actin was stained with phalloidin, and DNA was stained with 4',6-diamidino-2-phenylindole (DAPI). A minimum of 10 infected cells was imaged for each condition.

### Area of bacterial spread

To determine the efficiency of bacterial spread through monolayers, exponential phase *S*. *flexneri* were placed on cells seeded in 6-well plates at a multiplicity of infection (MOI, bacteria:cell) of 0.002–0.02. The plates were centrifuged at 830 *g* for 10 min to bring the bacteria into contact with the cells and were returned to 37°C for 1 hr, at which time gentamicin (25 μg/ml), which kills extracellular but not intracellular *Shigella*, was added to the media.

Monolayers were processed in one of two ways. For most experiments, monolayers were infected with GFP-expressing *S*. *flexneri*. These monolayers were imaged at approximately 16 hrs of infection using 4x magnification. For experiments in which rapamycin was used, the *Shigella* strains did not carry *gfp*; at 16 hrs of infection, infected monolayers were overlaid with 0.7% agarose in DMEM containing gentamicin and the vital dye neutral red and were then maintained at 37°C for an additional 24 hrs, at which time they were imaged on a plate scanner. For each condition, a minimum of 10–15 plaques was measured.

To measure bacterial spread in the presence of growth factor, plated cells were allowed to attach for 4 hr in 10% fetal bovine serum (vol/vol) and were then serum starved overnight in 1% fetal bovine serum, before proceeding with the infection as described above. At 1 hr of infection, in addition to gentamicin, insulin-like growth factor (100 ng/ml) was added to the media. The monolayers were imaged at 24 h of infection.

### Cell density in infected monolayers

Infection was performed with GFP-expressing *S*. *flexneri* essentially as described above for determining the area of bacterial spread, except that the MOI was 0.03 and infected cells were maintained with gentamicin, but not an agarose overlay. At 24 hr of infection, monolayers were stained with Hoechst (33342, Life Technologies) and were imaged using 4x and 10x magnification. Infectious foci were identified by GFP signal from the bacteria. The uninfected areas of the monolayer that were imaged were selected at random from within each quadrant of the well. Using iVision, 100 x 100 or 200 x 200 pixel boxes were drawn around infectious foci or the noninfectious monolayer, and the number of cells within each box was counted.

### Precipitation of bacterial proteins secreted by type III secretion

Plasmids that encode FLAG-tagged type III secreted effector proteins [47] were introduced into wild type *S*. *flexneri*. Each strain was grown to exponential phase, at which time IPTG was added to 0.1 mM to induce the expression of the FLAG-tagged effector. Cultures were returned to 37°C for 1 hr. Cultures were pelleted and resuspended in 30 mM HEPES (pH 7.7), 137 mM NaCl, 10 mM Congo red, and 0.1mM IPTG and incubated at 37°C for 30 mins. Culture supernatants were cleared by two serial centrifugation steps and were supplemented with 1 mM DTT, 1 mM EDTA, 1x Complete, 0.01% Triton X-100, and 300 mM NaCl. GST-IQGAP1, purified as described previously [45] and bound to glutathione sepharose beads, was incubated with cleared supernatants at 4°C for 2 hrs. Beads were recovered by centrifugation at 2,500 *g* for 5 min and washed three times with wash buffer (30 mM HEPES, pH 7.7, 300 mM NaCl, 0.01% Triton X-100). Bound proteins were eluted off of beads by boiling in SDS sample buffer. Proteins remaining in the cleared supernatants after incubation with IQGAP1 beads were precipitated with 15% trichloroacetic acid.

### Preparation of fusion proteins

GST-IQGAP1 was produced in *E*. *coli* and isolated using glutathione-Sepharose chromatography essentially as previously described [45]. Where indicated, IQGAP1 was further purified by cleaving GST using tobacco etch virus [48]. GST-OspB was produced and isolated as described for GST-IQGAP1. His-tagged OspB was purified with Ni^2+^-nitrilotriacetic acid agarose beads (Qiagen) following the manufacturer’s protocol. The size and purity of the GST- and His-tagged proteins were evaluated by SDS-PAGE and Coomassie staining. All proteins were at least 90% pure.

### In vitro binding assays

For assays with GST-tagged proteins, 3–5 μg purified untagged IQGAP1 was pre-incubated at 4°C for 1 h with 20 μl glutathione beads in 1 ml Buffer A (50 mM Tris-HCl, pH 7.4, 150 mM NaCl and 1% Triton X-100) containing 1X Protease & Phosphatase Inhibitor Cocktail (Thermo Scientific) and 1 mM PMSF. The beads were recovered by centrifugation, and the supernatant was transferred to tubes containing either 30 μl GST-OspB or 20 μl GST. All GST proteins were on glutathione-Sepharose beads. Samples were rotated for 3 h at 4°C, washed 5 times with Buffer A and resolved by SDS-PAGE. The gel was cut at ~100 kDa. The bottom portion was stained with Coomassie blue. The top portion was transferred to PVDF. Membranes were incubated for 1 h at 4°C with Blocking Buffer (LI-COR), then probed with anti-IQGAP1 polyclonal antibodies [45] overnight at 4°C. The membrane was incubated with infrared dye-conjugated (IRDye) anti-rabbit antibody for 1 h and antigen-antibody complexes were detected using the Odyssey Imaging System (LI-COR) as described [49].

For assays with His-tagged OspB, His-OspB was pre-cleared in Buffer A containing 20 μl glutathione-Sepharose beads as described in the prior paragraph for IQGAP1. The supernatants were transferred to tubes containing 20 μl GST-IQGAP1 on beads or 20 μl GST beads and incubated as above. Samples were processed by SDS-PAGE. The gel was cut at ~75 kDa. The top portion of the gel was stained with Coomassie Blue. The bottom portion was transferred to PVDF, probed with anti-His monoclonal antibody (Santa Cruz Biotechnology), followed by IRDye-conjugated anti-mouse antibody (LI-COR) and imaged as outlined above.

To identify the OspB binding site on IQGAP1, IQGAP1 proteins were generated by Transcription and Translation (TNT) kit (Promega). To prepare Transcend Biotin-Lysyl labeled IQGAP1 proteins, we mixed 40 μl TNT T7 Quick Master Mix, 1 μl 1 mM methionine, 1 μg pCDNA-IQGAP1 plasmid, 0.5–3 μl Transcend Biotin-Lysyl-tRNA, and added water to 50 μl. Samples were incubated at 30°C for 90 min. 40 μl GST-OspB or GST on glutathione-Sepharose beads was incubated with 20 μl TNT product in 1 ml Buffer B (Buffer A containing protease inhibitors and PMSF) and rotated at 4°C for 3 h. After 5 washes with Buffer A, samples were separated by 4–20% gradient SDS-PAGE and transferred to PVDF. Visualization was with Strep-HRP, Chemiluminescient Substrate, and exposure to X-ray film.

### Precipitation of proteins from mammalian cell lysates

MCF-7 human breast epithelial cells were grown to 90% confluence, washed twice with ice-cold PBS (155.6 mM NaCl, 1 mM KH_2_PO_4_, and 2.9 mM Na_2_HPO_4_, pH 7.4), and lysed with 500 ul of Buffer B. Lysates were processed by sonication for 10 s with a Model 100 Dismembrator (Fisher Scientific), and insoluble material was pelleted by centrifugation for 10 min. Rotation with glutathione-Sepharose beads for 1 h at 4°C was performed to pre-clear the supernatants. Equal amounts of protein lysate were incubated with 4 ug of GST or GST-OspB for 3 h at 4°C. After centrifugation, samples were washed five times with Buffer A and separated by SDS-PAGE. The gel was cut at 100-kDa. The bottom portion of the gel was stained with Coomassie blue. The upper portion was transferred to PVDF and probed with anti-IQGAP1 antibodies.

### Other western blot analyses

For all other western blot analysis, proteins were separated on SDS polyacrylamide gels, and western blot analysis was carried out using standard procedures and the following antibodies: FlagM2 (F1804, Sigma; diluted 1:200), IQGAP1 (ab33542, Abcam, Cambridge, MA; diluted to 1 μg/ml), phospho-S6 kinase (Thr-389) (9234, Cell Signaling Technology; diluted to 1:1000), total S6 kinase (2708, Cell Signaling Technology; diluted to 1:1000), phospho-Akt (Thr-308) (4056S, Cell Signaling Technology; diluted to 1:1000), phospho-Akt (Ser-473) (4060S, Cell Signaling Technology; diluted to 1:1000), pan-Akt (4691, Cell Signaling Technology; diluted to 1:1000), peroxidase-conjugated anti-beta actin (A3854, Sigma; diluted 1:10,000), and horseradish peroxidase conjugated goat anti-mouse secondary (Jackson; diluted 1:2000). Visualization was performed using SuperSignal West Pico Chemilumnescent Substrate or SuperSignal Femto Chemiluminescent Substrate (Thermo Fisher Scientific), per the manufacturer’s instructions. Densitometry of bands was performed using a Bio Rad Molecular Imager Chemi Doc XRS+ Imaging System and ImageJ software.

### Microscopy and data analysis

Microscopic images were collected using a Nikon Eclipse TE300 using the software IP Lab or iVision. For time lapse imaging to determine bacterial speed an image was taken every 5 sec for 15 min. Movies were compiled using Image J and the rate of bacterial movement was determined by tracking individual bacteria for 12 consecutive frames. For each experimental condition in each experiment, speeds were determined for 10 or more moving bacteria. The lengths of actin tails and bacterial protrusions were measured on still images using IP Lab.

## Supporting Information

S1 Text Supporting Information file. Contains supplemental methods and supplemental references.
(PDF)

S1 Table Cell characteristics and *S*. *flexneri* entry and actin based motility phenotypes are similar in the absence versus presence of IQGAP1 in MEFs. Comparison of cellular characteristics and *S*. *flexneri* actin-based virulence phenotypes IQGAP1^-/-^ versus IQGAP1^+/+^ MEFs. Each set of data is from a minimum of three independent experiments.
(PDF)

S1 Fig Depletion of IQGAP1 by siRNA leads to an increase in area of spread of *S*. *flexneri* through HeLa cell monolayers. siRNA knock-down of IQGAP1 in HeLa cells. (A) Western blot using antibody to IQGAP1. All lanes are from the same blot. Control siRNA targets GFP and had no effect on expression of bacterial GFP. Representative of three or more blots. (B) Area of spread of GFP-producing *S*. *flexneri* at 22 hrs. of infection. Data represent mean ± S.D. of three or more independent experiments. *, p = 0.03, Student’s two-tailed t test.
(TIF)

S2 Fig Actin and cadherin staining of IQGAP1^-/-^ and IQGAP1^+/+^ MEFs. (A) Phalloidin stained and DIC images of IQGAP1^-/-^ and IQGAP1^+/+^ MEFs. Representative of three or more independent experiments. (B) Immunofluorescence labeling with pan-cadherin antibody, staining with DAPI, and phase images of IQGAP1^-/-^ and IQGAP1^+/+^ MEFs. It is unclear why the nuclei give signal with the pan-cadherin antibody used. Arrows, cadherin at intercellular junctions. Representative of three independent experiments.
(TIF)

S3 Fig Density of cells at the edges of *S*. *flexneri*-infected foci in cell monolayers. Depiction of method used to measure cell density in infected monolayers. Placement of grids (dotted white box) at the edge of focus of infection with GFP-expressing *S*. *flexneri* (green, top panels) or randomly in uninfected areas of the monolayer (bottom panels). Cell nuclei stained with Hoechst (blue). Nuclei within boxes were counted.
(TIF)

S4 Fig Cell death and effect of rapamycin on proliferation of cells. (A) Cell death as measured by propidium iodide staining. Note that very few (less than 1%) cells lifted off under any condition. PI, propidium iodide. Representative of three independent experiments. (B) Proliferation rate of IQGAP1^-/-^ MEFs transiently transfected with p-OspB GFP or p-GFP and treated with rapamycin or DMSO carrier. Change in cell number (x 10^5^) as a function of time. (C) Representative images of cells on day 3 of experiment shown in panel B. Data represent the mean ± S.D. of three independent experiments.
(TIF)

S5 Fig Phosphorylation of Akt. (A) Inhibition of phosphorylation of AKT (T308) by PI 3-kinase (PI3K) inhibitor LY294002 during *S*. *flexneri* infection does not block activation of mTORC1, since S6K phosphorylation is not inhibited. (B) Phosphorylation of AKT at Ser-473 is similar in the presence or absence of OspB and IQGAP1. Phospho-Akt Ser-473 and total Akt in IQGAP1^-/-^ versus IQGAP1^+/+^ MEFs transiently transfected with OspB GFP or GFP alone. Western blots. Data are representative of three independent experiments.
(TIF)

S6 Fig Time course of mTor activation during *S*. *flexneri* infection of various cell lines. (A) Time course of S6K phosphorylation in MEFs, HeLa cells, and Caco2 cells infected with WT *S*. *flexneri*. Western blot representative of three independent experiments. Apparent MWs are indicated in Kd. -, no infection. (B) Growth curves of *S*. *flexneri* strains *in vitro* in the presence or absence of 10 nM rapamycin. Data are from one experiment that is rrepresentive of three independent experiments.
(TIF)

S7 Fig Model of IQGAP1 serving as a scaffold for OspB-dependent activation of mTORC1, which causes cell proliferation and leads to smaller areas of *S*. *flexneri* spread through monolayers. The secreted *Shigella* effector protein OspB interacts with the IQ region of IQGAP1, adjacent to the WW region, to which mTOR binds [17]. OspB activation of mTORC1 induces increased cell proliferation around foci of infection, leading to net smaller areas of spread through the monolayer. In the two cases depicted, bacteria are spread through the same number of viable cell layers (two, arbitrarily chosen), and bacterial numbers within the infectious foci are similar. OspB activation of mTORC1 is blocked by rapamycin.
(TIF)
