# Supplementary material for: Shigella Effector OspB Activates mTORC1 in a Manner That Depends on IQGAP1 and Promotes Cell Proliferation
Source: PLoS Pathog. 2015 Oct 16;11(10):e1005200. doi: 10.1371/journal.ppat.1005200 (PMC4608727; doi:10.1371/journal.ppat.1005200)
Supplement: S1 Text — Contains supplemental methods and supplemental references. (PDF) [file ppat.1005200.s001.pdf]

Supplemental Methods

Bacterial strains, plasmids, growth conditions. Wild-type *S. flexneri* serotype 2a strain 2457T (Labrec et al., 1964), an isogenic *ospB* deletion mutant strain (gift from C. Lesser), and an isogenic non-invasive strain, BS103, which is cured of the virulence plasmid (Maurelli et al., 1984), were grown in tryptic soy broth from individual colonies that were red on agar containing Congo red. IQGAP1 expression plasmids have been described (Ho et al., 1999). For bacterial expression, *ospB* was cloned under the control of the native promoter in pACYC184, and for mammalian expression, into pcDNA3 as a transcriptional fusion to *gfp*. Each of 26 *S. flexneri* type III secreted effector proteins were tagged at the C-terminus with FLAG and expressed under the control of the impaired Tac promoter in pDSW206 (Weiss et al., 1999), as described (Costa et al., 2012).

Cells. HeLa cells (ATCC), MEFs, and Caco2 cells were maintained in Dulbecco's modified media (DMEM) supplemented with fetal bovine serum (10% vol/vol). MEFs isolated from IQGAP1$^{-/-}$ and littermate control IQGAP1$^{+/+}$ mice have been described (Ren et al., 2007). Immunofluorescence labeling of cadherin was with pan-cadherin antibody (Sigma C3678) following fixation with methanol.

Western blot analysis. Proteins were separated on SDS polyacrylamide gels, and western blot analysis was carried out using standard procedures and the following antibodies: anti-FlagM2 (F1804, Sigma; diluted 1:200), anti-IQGAP1 (ab33542, Abcam, Cambridge, MA; diluted to 1 mg/ml), anti-phospho-S6 kinase

(Thr-389) (9234, Cell Signaling Technology; diluted to 1:1000) anti-total S6 kinase (2708, Cell Signaling Technology; diluted to 1:1000), anti-phospho-4EBP1 (Thr-37/46) (2855, Cell Signaling Technology; diluted to 1:1000), anti-total 4EBP1 (9452S, Cell Signaling Technology; diluted to 1:1000), anti-phospho-Akt (Thr-308) (4056S, Cell Signaling Technology; diluted to 1:1000), anti-phospho-Akt (Ser-473) (4060S, Cell Signaling Technology; diluted to 1:1000), peroxidase-conjugated anti-beta actin (A3854, Sigma; diluted 1:10,000) and horseradish peroxidase conjugated goat anti-mouse secondary (Jackson; diluted 1:2000). Visualization was performed using SuperSignal West Pico Chemilumnescent Substrate or SuperSignal Femto Chemiluminescent Substrate (Thermo Fisher Scientific), per the manufacturer’s instructions. Densitometry of bands was performed using a Bio Rad Molecular Imager Chemi Doc XRS+ Imaging System and ImageJ software.

Data analysis and statistical methods. All data are from three or more independent experiments. Differences between data sets were determined using standard statistical methods.

Supplemental references

Costa, S.C., Schmitz, A.M., Jahufar, F.F., Boyd, J.D., Cho, M.Y., Glicksman, M.A., and Lesser, C.F. (2012). A new means to identify type 3 secreted effectors: functionally interchangeable class IB chaperones recognize a conserved sequence. MBio 3.

Ho, Y.D., Joyal, J.L., Li, Z., and Sacks, D.B. (1999). IQGAP1 integrates Ca2+/calmodulin and Cdc42 signaling. J Biol Chem 274, 464-470.

Labrec, E.H., Schneider, H., Magnani, T.J., and Formal, S.B. (1964). Epithelial Cell Penetration as an Essential Step in the Pathogenesis of Bacillary Dysentery. Journal of bacteriology 88, 1503-1518.

Maurelli, A.T., Blackmon, B., and Curtiss, R., 3rd (1984). Loss of pigmentation in Shigella flexneri 2a is correlated with loss of virulence and virulence-associated plasmid. Infect Immun 43, 397-401.

Ren, J.G., Li, Z., and Sacks, D.B. (2007). IQGAP1 modulates activation of B-Raf. Proc Natl Acad Sci U S A 104, 10465-10469.

Weiss, D.S., Chen, J.C., Ghigo, J.M., Boyd, D., and Beckwith, J. (1999). Localization of FtsI (PBP3) to the septal ring requires its membrane anchor, the Z ring, FtsA, FtsQ, and FtsL. J Bacteriol 181, 508-520.
