# Supplementary material for: Shigella Effector OspB Activates mTORC1 in a Manner That Depends on IQGAP1 and Promotes Cell Proliferation
Source: PLoS Pathog. 2015 Oct 16;11(10):e1005200. doi: 10.1371/journal.ppat.1005200 (PMC4608727; doi:10.1371/journal.ppat.1005200)
Supplement: S1 Table — Comparison of cellular characteristics and S. flexneri actin-based virulence phenotypes IQGAP1-/- versus IQGAP1+/+ MEFs. Each set of data is from a minimum of three independent experiments. (PDF) [file ppat.1005200.s002.pdf]

Table S1. Cell characteristics and *S. flexneri* entry and actin based motility phenotypes are similar in the absence versus presence of IQGAP1 in MEFs

		Measure	Data (mean ± S.D.)	
			IQGAP1$^{-/-}$	IQGAP1$^{+/+}$
S. flexneri virulence phenotypes	Entry	Internalized bacteria (no./well)	1520 ± 520	1460 ± 590
		Plaque formation (no./well)	58 ± 23	49 ± 26
	Actin based motility [a]	Actin tail length (μm)	29 ± 7	28 ± 4
		Actin tail frequency (% of bacteria)	44 ± 17	51 ± 16
		Speed in the cytoplasm (μm/sec)	50 ± 21	56 ± 28
		Speed in protrusions (μm/sec)	42 ± 21	43 ± 17
Cellular phenotypes	Cell size	Area (pixels x 10^3)	8.1 ± 3.0	7.2 ± 2.0
	Cortical actin density	Signal intensity at cortex/signal intensity in cell body (a.u.)	0.92 ± 0.04	0.89 ± 0.03

[a] Measurements were taken at 2 hrs. of infection.
