# Supplementary figures and images for: Shigella Effector OspB Activates mTORC1 in a Manner That Depends on IQGAP1 and Promotes Cell Proliferation
Source: PLoS Pathog. 2015 Oct 16;11(10):e1005200. doi: 10.1371/journal.ppat.1005200 (PMC4608727; doi:10.1371/journal.ppat.1005200)

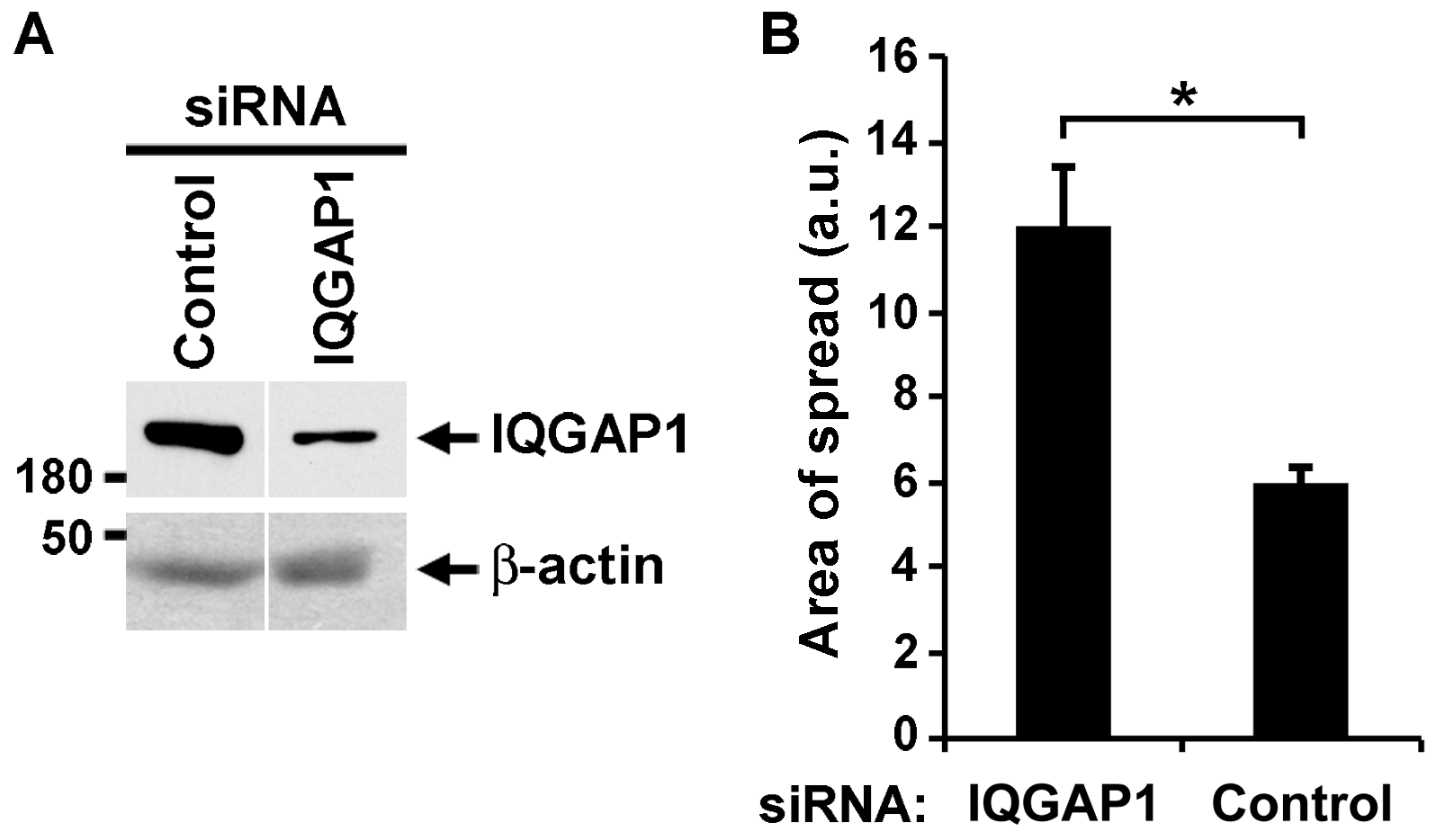

Supplement: S1 Fig — siRNA knock-down of IQGAP1 in HeLa cells. (A) Western blot using antibody to IQGAP1. All lanes are from the same blot. Control siRNA targets GFP and had no effect on expression of bacterial GFP. Representative of three or more blots. (B) Area of spread of GFP-producing S. flexneri at 22 hrs. of infection. Data represent mean ± S.D. of three or more independent experiments. *, p = 0.03, Student’s two-tailed t test. (TIF) [file ppat.1005200.s003.tif]

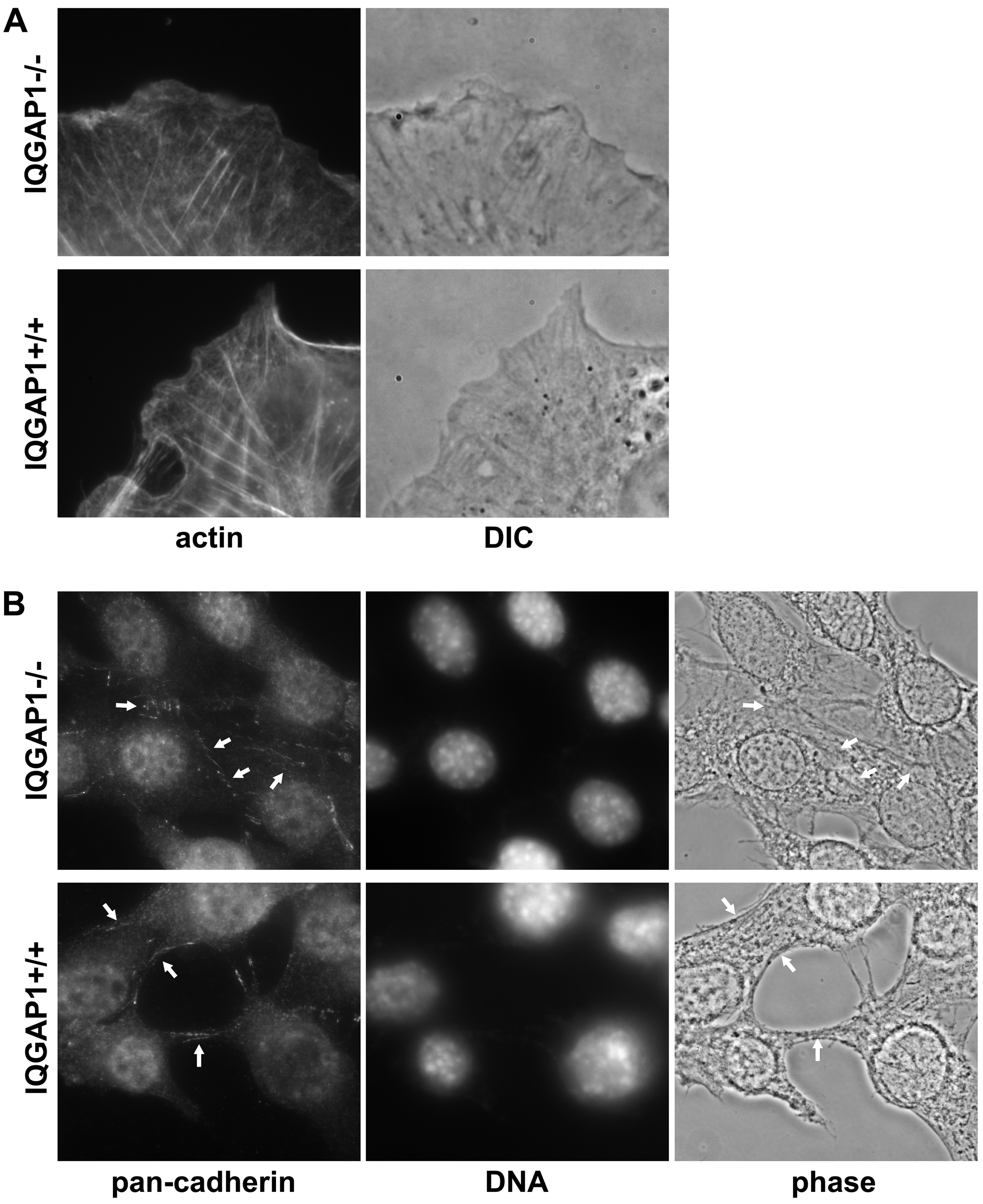

Supplement: S2 Fig — (A) Phalloidin stained and DIC images of IQGAP1-/- and IQGAP1+/+ MEFs. Representative of three or more independent experiments. (B) Immunofluorescence labeling with pan-cadherin antibody, staining with DAPI, and phase images of IQGAP1-/- and IQGAP1+/+ MEFs. It is unclear why the nuclei give signal with the pan-cadherin antibody used. Arrows, cadherin at intercellular junctions. Representative of three independent experiments. (TIF) [file ppat.1005200.s004.tif]

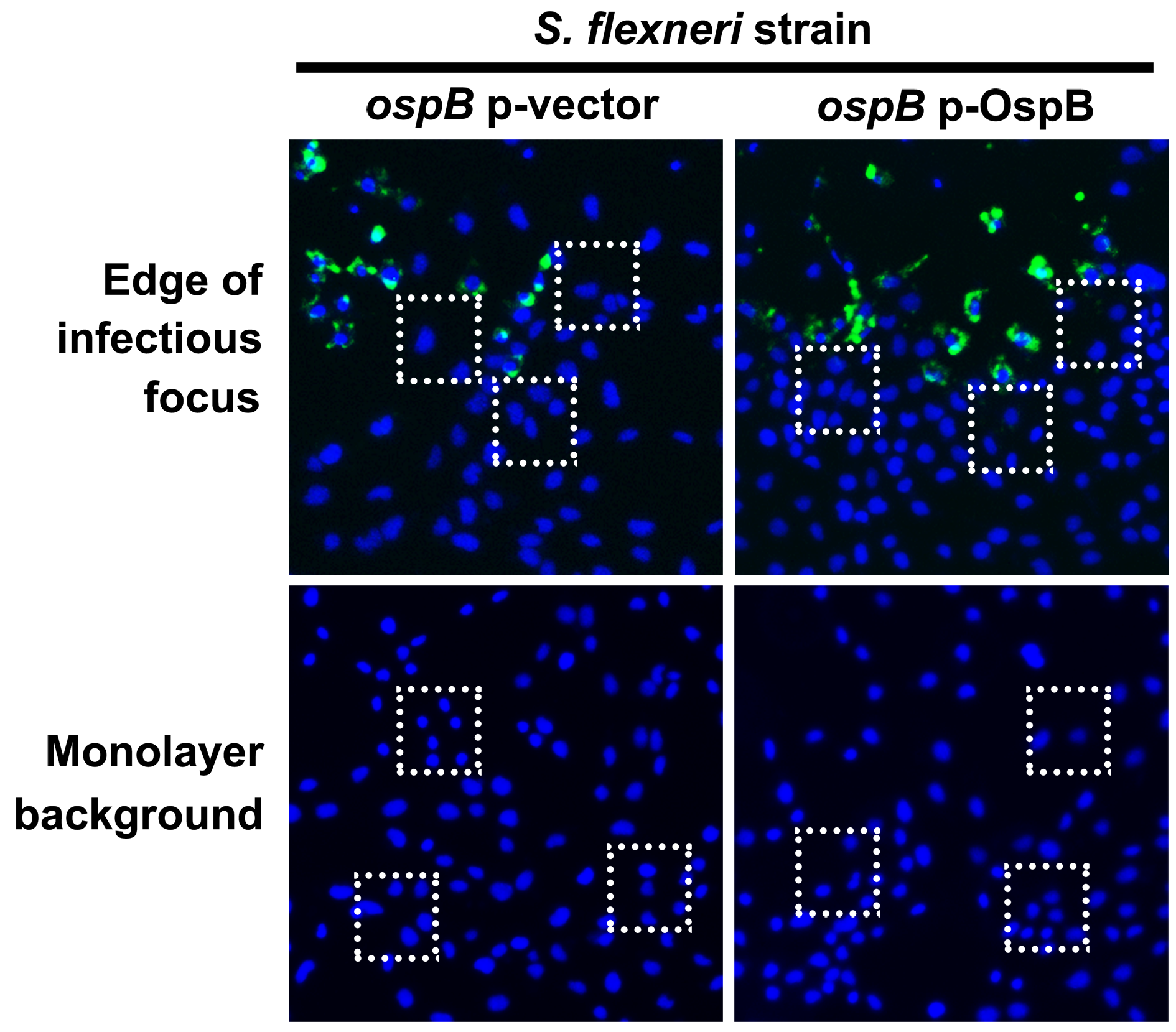

Supplement: S3 Fig — Depiction of method used to measure cell density in infected monolayers. Placement of grids (dotted white box) at the edge of focus of infection with GFP-expressing S. flexneri (green, top panels) or randomly in uninfected areas of the monolayer (bottom panels). Cell nuclei stained with Hoechst (blue). Nuclei within boxes were counted. (TIF) [file ppat.1005200.s005.tif]

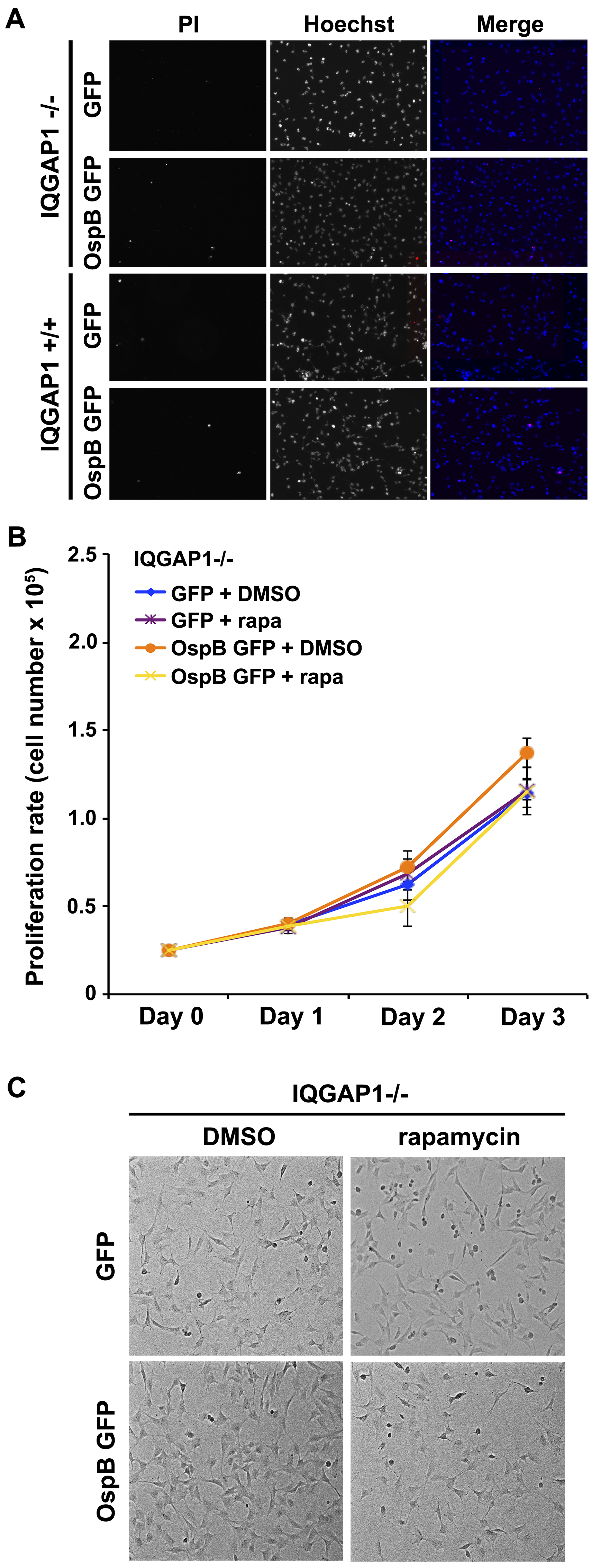

Supplement: S4 Fig — (A) Cell death as measured by propidium iodide staining. Note that very few (less than 1%) cells lifted off under any condition. PI, propidium iodide. Representative of three independent experiments. (B) Proliferation rate of IQGAP1-/- MEFs transiently transfected with p-OspB GFP or p-GFP and treated with rapamycin or DMSO carrier. Change in cell number (x 105) as a function of time. (C) Representative images of cells on day 3 of experiment shown in panel B. Data represent the mean ± S.D. of three independent experiments. (TIF) [file ppat.1005200.s006.tif]

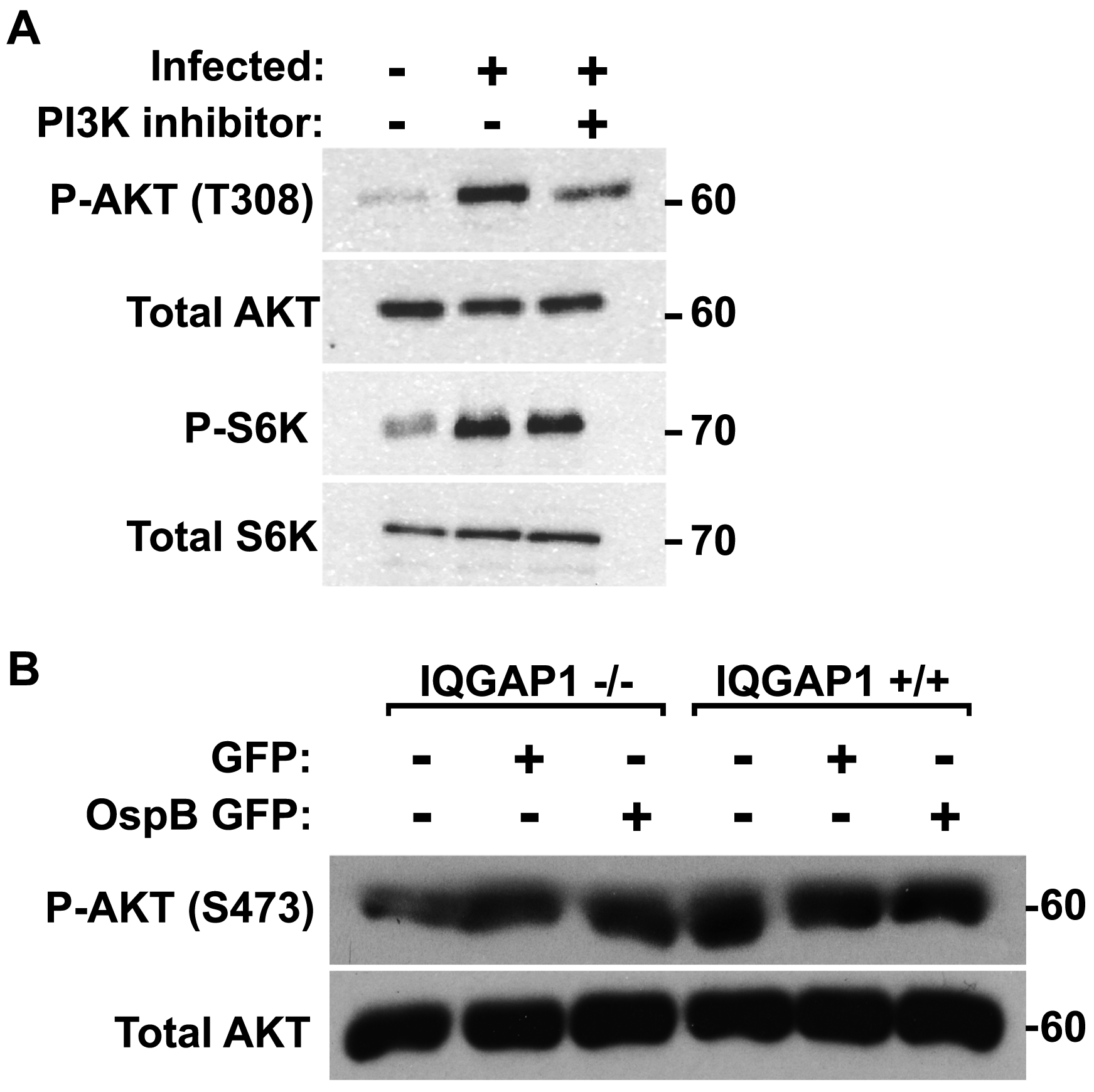

Supplement: S5 Fig — (A) Inhibition of phosphorylation of AKT (T308) by PI 3-kinase (PI3K) inhibitor LY294002 during S. flexneri infection does not block activation of mTORC1, since S6K phosphorylation is not inhibited. (B) Phosphorylation of AKT at Ser-473 is similar in the presence or absence of OspB and IQGAP1. Phospho-Akt Ser-473 and total Akt in IQGAP1-/- versus IQGAP1+/+ MEFs transiently transfected with OspB GFP or GFP alone. Western blots. Data are representative of three independent experiments. (TIF) [file ppat.1005200.s007.tif]

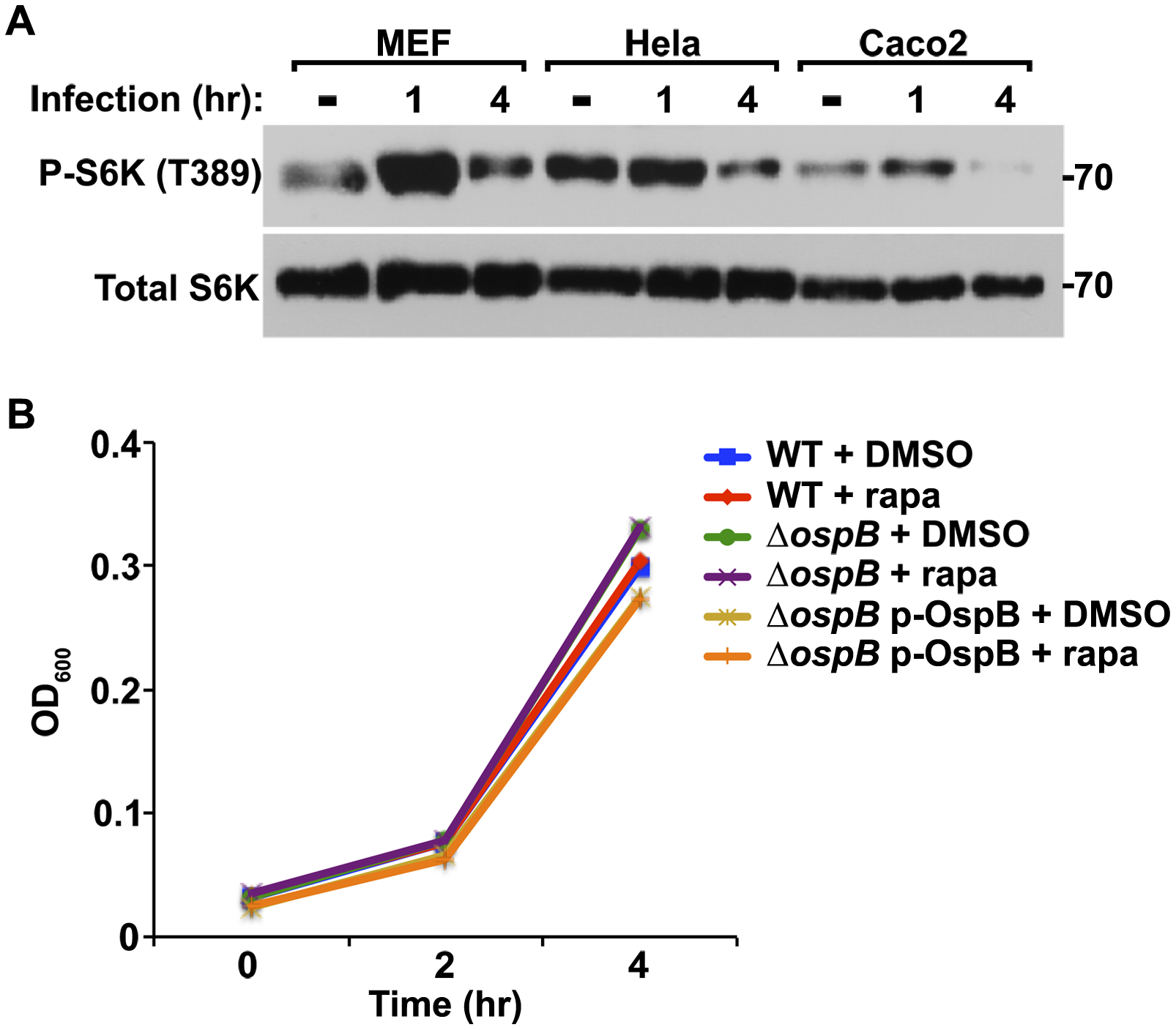

Supplement: S6 Fig — (A) Time course of S6K phosphorylation in MEFs, HeLa cells, and Caco2 cells infected with WT S. flexneri. Western blot representative of three independent experiments. Apparent MWs are indicated in Kd. -, no infection. (B) Growth curves of S. flexneri strains in vitro in the presence or absence of 10 nM rapamycin. Data are from one experiment that is rrepresentive of three independent experiments. (TIF) [file ppat.1005200.s008.tif]

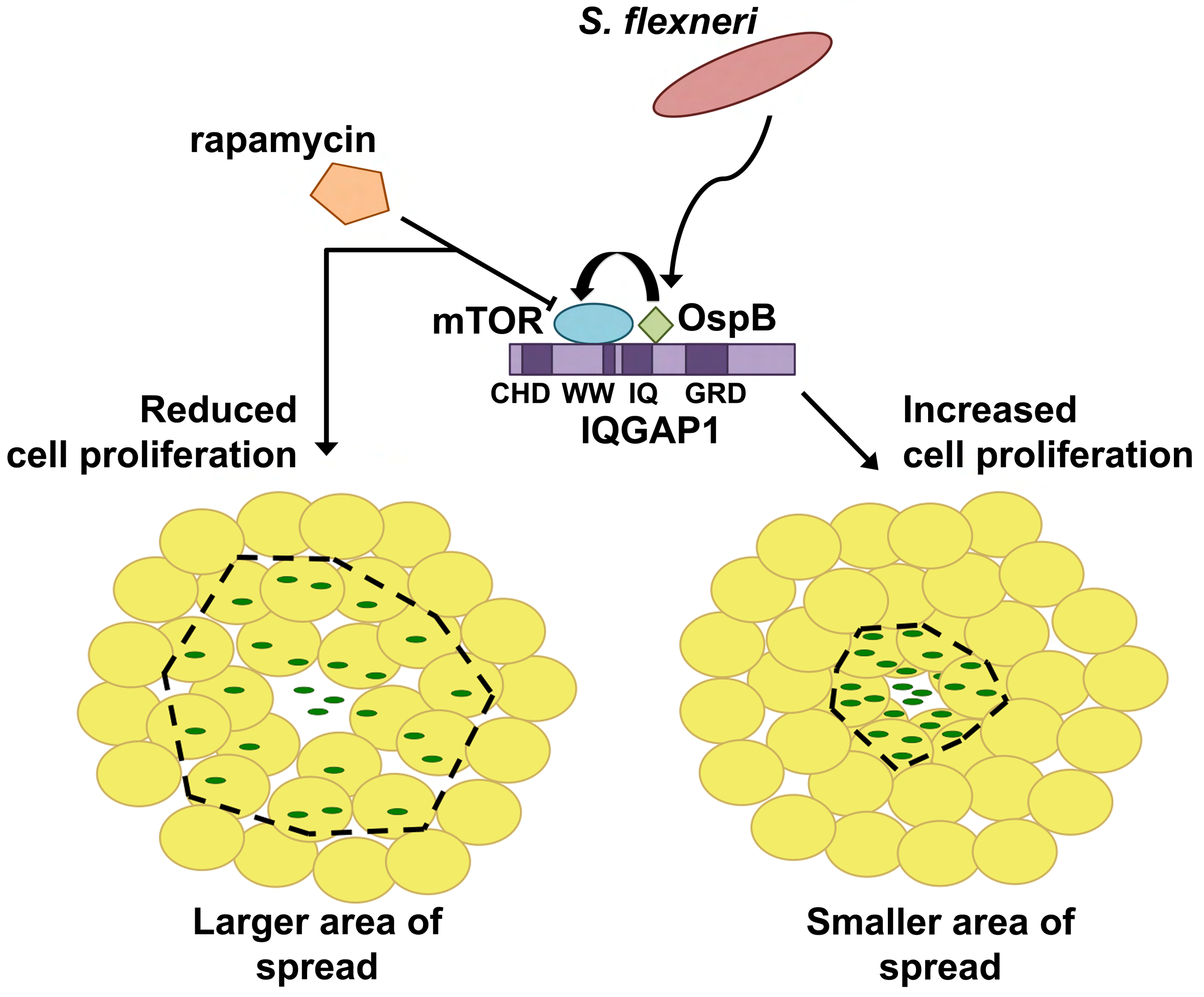

Supplement: S7 Fig — The secreted Shigella effector protein OspB interacts with the IQ region of IQGAP1, adjacent to the WW region, to which mTOR binds [17]. OspB activation of mTORC1 induces increased cell proliferation around foci of infection, leading to net smaller areas of spread through the monolayer. In the two cases depicted, bacteria are spread through the same number of viable cell layers (two, arbitrarily chosen), and bacterial numbers within the infectious foci are similar. OspB activation of mTORC1 is blocked by rapamycin. (TIF) [file ppat.1005200.s009.tif]
